# An Insect Salivary Sheath Protein Triggers Plant Resistance to Insects and Pathogens as a Conserved HAMP

**DOI:** 10.1002/advs.202415474

**Published:** 2025-04-01

**Authors:** Liangxuan Qi, Jing Li, Shuai Li, Jing Li, Han Wang, Lei Yang, Xinyang Tan, Zhichang Zhao, Guanghua Luo, Maofeng Jing, Ary A. Hoffmann, Jichao Fang, Rui Ji

**Affiliations:** ^1^ Institute of Plant Protection Jiangsu Academy of Agricultural Sciences Jiangsu Key Laboratory for Food and Safety‐State Key Laboratory Cultivation Base of Ministry of Science and Technology Nanjing 210014 China; ^2^ College of Plant Protection Nanjing Agricultural University Nanjing 210014 China; ^3^ School of BioSciences Bio21 Institute University of Melbourne Parkville VIC 3010 Australia

**Keywords:** HAMP, myosin, plant defense, rice planthopper, salivary sheath

## Abstract

Herbivore‐associated molecular patterns (HAMPs) in saliva enable plants to detect herbivores and activate pattern‐triggered immunity (PTI). Piercing‐sucking herbivores secrete gel saliva, forming salivary sheaths that assist in feeding, however, the role of proteins within these sheaths in modulation of plant defenses remains poorly understood. Here, a thermostable HAMP, myosin light chain 1‐like (myosin) is identified, from the salivary sheath of the small brown planthopper (SBPH) Laodelphax striatellus. Myosin is a widely conserved arthropod protein, and acts as an elicitor of BAK1‐dependent PTI responses in several plant species. Plants are able to specifically recognize the myosin 41‐amino‐acid peptide (MP41), which acts as a minimal immunogenic epitope. Furthermore, myosin and MP41 stimulate jasmonic acid and H_2_O_2_ production in rice. The resulting defenses not only diminish planthopper performance directly but also induce volatile emissions, attracting a common parasitoid. Additionally, expression of myosin in rice increased plant resistance to a chewing insect as well as to viral and fungal pathogens. However, silencing *myosin* in SBPH resulted in disruption of salivary sheath formation, reducing insect feeding efficiency. This study demonstrates that myosin from the SBPH salivary sheath serves as a critical and unavoidable HAMP, triggering broad‐spectrum plant resistance to various insects and pathogens.

## Graphical abstract

The salivary sheath protein myosin from SBPH is critical for the formation of the salivary sheath and feeding. However, myosin functions as a HAMP and triggered plant BAK1‐mediated PTI responses, which include the activation of calcium signaling pathways, MAPK phosphorylation, ROS bursts, and cell death, thereby triggering JA pathway. The minimal immunogenic peptide, MP41, is characterized. The resulting defenses not only diminish planthopper performance directly but also induce volatile emissions in rice, attracting a common parasitoid. Moreover, myosin enhances the rice's broad‐spectrum resistance to various insects and pathogens. Additionally, myosin proteins are conserved across arthropods and induced defense response in multiple plant species.

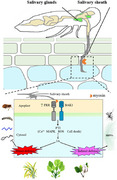

## 1 Introduction

Over millions of years of evolution, plants have developed an intricate immune system to defend against both pathogens and herbivores. Pattern recognition receptors (PRRs) embedded in plant cell membranes detect pathogen/herbivore‐associated molecular patterns (PAMPs/HAMPS) in the apoplasts and enable plants to activate pattern‐triggered immunity (PTI), such as increases in cytosolic calcium ion (Ca^2+^) concentrations, activation of mitogen‐activated protein kinases (MAPKs), and a burst of reactive oxygen species (ROS). These early signaling events trigger defense‐related phytohormone pathways, particularly those involving jasmonic acid (JA) and salicylic acid (SA). The activation of these pathways results in the production of defensive compounds, thereby enhancing plant resistance.^[^ ^1^ ^]^

Co‐receptors of PRRs, such as BRI1‐associated kinase 1 (BAK1) and suppressor of BIR1‐1 (SOBIR1), are known to play crucial roles in modulating PTI signaling and initiating basal immune responses to pathogens. Several lines of evidence suggest these co‐receptors also significantly contribute to herbivore resistance. For example, the *Arabidopsis* BAK1 enhances plant resistance to aphids by inducing ROS accumulation and callose deposition.^[^ ^2^ ^]^ PAMPs and their immunogenic peptides have been well characterized in various pathogens, such as the highly conserved 22‐amino acid fragment of flagellin (flg22), the 18‐amino acid domain of EF‐Tu (elf18), and a conserved 22‐amino acid peptide from Ser‐Thr‐rich glycosylphosphatidylinositol‐anchored protein.^[^ ^3^, ^4^ ^]^ Compared to the extensive research on PAMPs from plant pathogens, studies on HAMPs from herbivorous insects have been relatively limited, although a few elicitors have also been identified in piercing‐sucking insects, including cathepsin B3, protein disulfide isomerase (PDI), NlMLP, NlVgN, and NlG14.^[^ ^5^, ^6^, ^7^, ^8^, ^9^, ^10^ ^]^ The first HAMP in piercing‐sucking insects recognized by plant PRRs capable of activating PTI responses has been recently identified in the mirid bug *Riptortus pedestris*.^[^ ^11^ ^]^ Salivary RPH1 induces PTI responses in *Nicotiana benthamiana*, requiring the co‐receptors BAK1 and SOBIR1, although its immunogenic peptide and the receptor remain unidentified.^[^ ^11^ ^]^ Additionally, several HAMPs have been identified from the oral secretions of chewing insects.^[^ ^12^, ^13^ ^]^ The first plant PRR capable of recognizing HAMP from chewing insects has been recently identified. A leucine‐rich repeat receptor of the HAMP inceptin confers inceptin‐induced responses and enhances plant defense against armyworms (*Spodoptera exigua*).^[^ ^14^ ^]^ Some particular components in the oral secretions of *Mythimna loreyi* may be recognized by the rice receptors PLANT ELICITOR PEPTIDE RECEPTOR 1 (PEPR1), CHITIN ELICITOR RECEPTOR KINASE 1 (CERK1) and CHITIN ELICITOR‐BINDING PROTEIN (CEBiP), triggering defense responses in rice.^[^ ^15^, ^16^ ^]^ As a result, the mechanisms by which plants perceive herbivores and trigger PTI are still largely unexplored.

In addition to PTI, plants have evolved nucleotide‐binding leucine‐rich repeat (NLR) receptors that specifically recognize cytoplasmic effectors, leading to effector‐triggered immunity (ETI) and increased specific resistance. Key modulators of ETI signaling, such as suppressor of the G2 allele of skp1 (SGT1) and heat shock protein 90 (HSP90), stabilize NLR proteins. Non‐race‐specific disease resistance 1 (NDR1) and enhanced disease susceptibility 1 (EDS1) also participate in NLR‐mediated signaling.^[^ ^10^ ^]^ These ETI‐related signaling components influence plant‐herbivore interactions. For instance, SGT1 and HSP90 are vital for Mi‐1‐mediated resistance to aphids and whiteflies in tomato (*Solanum lycopersicum*).^[^ ^17^ ^]^ Notably, PDI from *Tetranychus evansi* induces plant defense in *N. benthamiana*, requiring SGT1 and HSP90 for ETI.^[^ ^10^ ^]^

Sap‐sucking insects use their stylets to penetrate deeply into the phloem and extract nutrients from the sap. During this process, they secrete both watery and gel saliva from their salivary glands into plant tissues. The gel saliva solidifies to form salivary sheaths, primarily in the intercellular spaces of plants. Such sheath encases the full length of the stylet, providing mechanical stability and effectively sealing the penetration site.^[^ ^18^ ^]^ In aphids and planthoppers, inhibiting the expression of structural sheath proteins disrupts the formation of the salivary sheath, impairing feeding from plant sieve tubes.^[^ ^7^, ^19^ ^]^ While effectors from insect watery saliva are well‐documented, current research into the salivary sheath has largely focused on its mechanical function, with its role in modulating plant defense remaining mostly unexplored.

The small brown planthopper (SBPH), Laodelphax striatellus, is a major pest of rice, causing extensive yield losses both directly through feeding and indirectly by transmitting viral diseases including rice stripe virus (RSV).^[^ ^20^ ^]^ Like many sap‐sucking insects, planthoppers secrete a mixture of watery and gel saliva during feeding, with several salivary proteins involved in the formation of salivary sheaths.^[^ ^7^ ^]^ However, the roles of only two salivary sheath proteins in modulating plant defense have recently been characterized. One, a mucin‐like protein (NlMLP) has been identified as an elicitor in contributing to both salivary sheath formation and the activation of plant immune responses.^[^ ^7^ ^]^ In contrast, the effector LsSP1, which is located in the plant apoplast, is not essential for salivary sheath formation, but significantly diminishes activation of plant defenses by NlMLP, benefiting planthopper performance on rice (*Oryza sativa*).^[^ ^21^ ^]^ The plant apoplast space serves as a critical battleground between the host and pathogens and/or herbivores. The insect salivary sheath, which primarily forms in the plant apoplast, comprises various proteins that directly contact plant cells^[^ ^19^, ^21^ ^]^ and may be recognized by PRRs as HAMPs, activating PTI in host plants.

In this study, we identified the proteins present in the salivary sheaths of SBPH and transiently expressed them in *N. benthamiana* leaves. Of these proteins, myosin light chain 1‐like (myosin) was found to induce cell death and ROS bursts. Myosins consist of two heavy chains and several light chains, including myosin light chain 1. They serve as molecular motors, transporting a variety of cargo along actin filaments, and are essential components of myofibrillar filaments. As part of the cytoskeleton, myosins generate the forces required for cytoplasmic flow, organelle movement, material transport, mitosis, cytoplasmic division, and apical growth in cells. They also play crucial roles in physiological processes such as phagocytosis, cell motility, immunity, and nutrient absorption.^[^ ^22^ ^]^ For example, rat myosin light chain 4 (MYL4) regulates autophagic flux in atrial cardiomyocytes by affecting lysosomal mobility.^[^ ^23^ ^]^ *Arabidopsis* myosin XIK is part of the BIK1 interactome, and functions as a molecular scaffold, facilitating rapid and robust PTI responses.^[^ ^24^, ^25^ ^]^ Previous research has revealed the presence of myosin in planthopper saliva,^[^ ^26^ ^]^ however, there are currently no reports on the role of the structural protein myosins in arthropod salivary sheath formation or in the regulating host immune responses.

In this study, we focused on the function of the salivary sheath protein myosin from SBPH as a conserved HAMP. Myosin triggered PTI responses in several plant species. Its minimal immunogenic peptide specifically recognized by the plants comprised 41 amino acids (MP41), and activated defense responses. These defense responses ultimately enhanced resistance against sap‐sucking and chewing insects, as well as viral and fungal pathogens. Since myosin is crucial for the formation of the planthopper salivary sheath, it appears unlikely that planthoppers can evade this recognition. Our findings suggest that certain plants can identify an insect salivary sheath protein to activate PTI, and thus resist multiple forms of pest damage. This research provides new insights into insect‐plant interactions and proposes potential strategies for broad‐spectrum pest management.

## 2 Results

### 2.1 The Salivary Sheath Protein Myosin Is Essential for Salivary Sheath Formation and Feeding Performance in SBPH

An important step in understanding the functions of different proteins of the salivary sheath in the interaction between SBPH and host rice is to identify those components. We therefore analyzed salivary sheath proteins using liquid chromatography‐tandem mass spectrometry (LC‐MS/MS). A total of 42 proteins were identified, of which 19 with 2 or more unique peptides (high reliability of sequencing) were detected (Table S1, Supporting Information). In order to screen for candidate SBPH elicitors, we expressed these 19 proteins transiently in *N. benthamiana* leaves, resulting in the identification of two structural proteins (myosin and actin‐related protein 1) that induced cell death and ROS production (Figure S1, Supporting Information). A BLAST search was conducted using the rice database on NCBI, and we found that there is a high homology (89% identity) of actin‐related protein 1 and a low homology (36% identity) of myosin between SBPH and rice, and we therefore focused on myosin rather than the actin‐related protein for further study.

**Figure 1:**
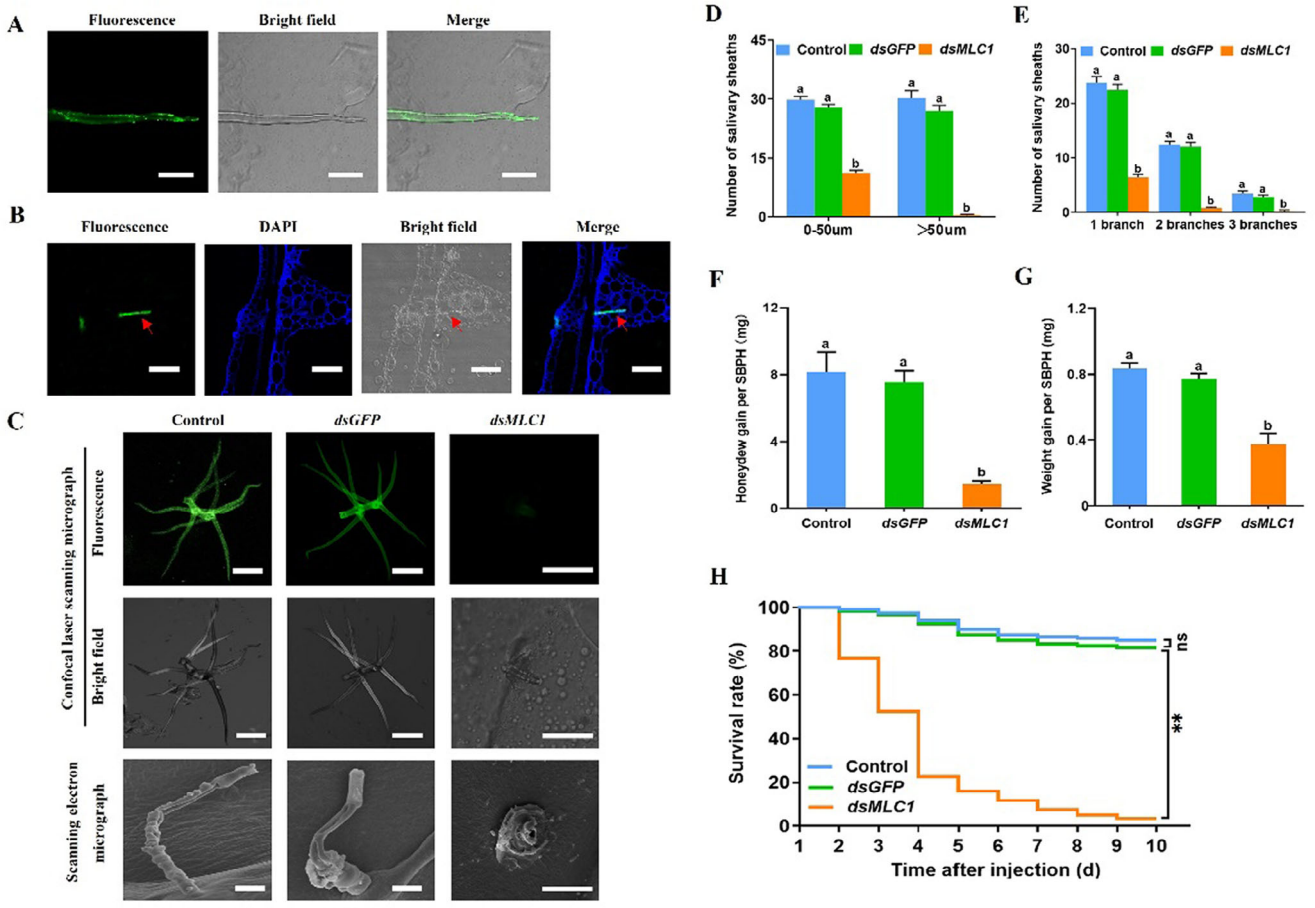
Myosin is a salivary sheath protein and is essential for SBPH feeding. (A–B) Immunohistochemical staining of myosin in the salivary sheath of SBPH fed with an artificial diet (A) and on rice stem (B). The salivary sheaths were incubated with anti‐myosin serum conjugated with Alexa Fluor 488 NHS Ester (green) and examined using a confocal scanning microscope. The nucleus was stained with DAPI (blue), and the red arrows indicate the salivary sheaths. The SBPH nymphs were fed on an artificial diet in Parafilm sachets for 48 h, and the salivary sheaths were collected and observed. (C) Effects of *myosin* silencing on salivary sheath formation. Confocal scanning micrographs and scanning electron micrographs showing the myosin staining and the morphology of SBPH salivary sheaths in insects fed with an artificial diet. Green, myosin. Fourth‐instar nymphs were injected either with *myosin* dsRNA (*dsmyosin*) or *GFP* dsRNA (*dsGFP*), with the control group consisting of untreated SBPH. Bars = 20 µm. (D–E) Distribution (+ SE, *n* = 5) of branch number (D) and length (E) of SBPH salivary sheaths formed on each Parafilm sachet. Five Parafilm sachets were selected from each group and counted under a light microscope, with each Parafilm sachet containing the artificial diet for 20 fourth‐instar nymphs. (F–G) Mean amount of honeydew excretion (+ SE, *n* = 10; F) and mean weight gain (+ SE, *n* = 10; G) per newly emerged female SBPH were calculated. Different letters indicate significant differences among treatments (P < 0.05, one‐way ANOVA followed by Duncan's multiple range test for D–G). (H) Survival curves (*n* = 6) of *dsmyosin ‐*SBPH, *dsGFP*‐SBPH, or control groups on rice. Kaplan–Meier tests were used for determining significant survival differences in H, **p < 0.01; ns, not significant.

*Myosin* encodes a protein of 161 amino acids that lacks a signal peptide and transmembrane domain, but is a potential unconventionally secreted protein (Figure S2, Supporting Information). *Myosin* was expressed at all developmental stages of SBPH (Figure S3, Supporting Information) but was highly expressed in the salivary glands compared to the gut, fat body, ovary, testis, and carcass (Figure S3, Supporting Information). Immunohistochemistry staining confirmed the presence of myosin on the surface of the salivary sheaths of both SBPH fed with an artificial diet in Parafilm sachets and those fed on rice plants (**Figure** **1**A,B).

To investigate the impact of this protein on salivary sheath formation, we synthesized a double‐stranded RNA targeting *myosin* (*dsmyosin*) and injected it into SBPH nymphs to induce RNA interference (RNAi). This treatment effectively reduced *myosin* transcript levels by 81–96% over 6 days (2 to 8 days post‐injection) (Figure S4, Supporting Information). We then observed the salivary sheaths of SBPH fed on an artificial diet in Parafilm sachets using fluorescence microscopy and scanning electron microscopy. The fluorescence microscopy analysis revealed that almost no fluorescence signal of myosin was detected in salivary sheath from SBPHs injected with *dsmyosin* (*dsmyosin*‐SBPHs), while clear fluorescence signals were obvious in those of control SBPHs receiving no injection (Control) and those injected with *dsGFP* (*dsGFP*‐SBPHs, Figure 1C). Moreover, *dsmyosin*‐SBPHs produced significantly shorter and less branched salivary sheaths compared to those produced by the *dsGFP*‐SBPH insects and control groups (Figure 1D,E). Scanning electron microscopy further showed that the structures of the salivary sheaths secreted by *dsmyosin*‐SBPHs were incomplete and predominantly amorphous, while those secreted by control and *dsGFP*‐SBPHs groups had complete and typical structures (Figure 1C). Thus, myosin is essential for proper salivary sheath formation.

Since the salivary sheath is critical for insect feeding, we also investigated whether silencing *myosin* affected SBPH feeding, body weight, and survival on rice. We found that silencing *myosin* significantly reduced honeydew excretion by SBPHs (Figure 1F). Furthermore, *dsmyosin*‐SBPHs exhibited lower weight gain (Figure 1G) and survival rates compared to the *dsGFP*‐SBPHs and control groups (Figure 1H). Collectively, these findings indicate that silencing *myosin* in SBPHs resulted in defective and short salivary sheaths, adversely affecting feeding and overall insect performance.

### 2.2 Myosin‐Triggered Cell Death and ROS Production Is Not Affected by Heat Treatment

**Figure 2:**
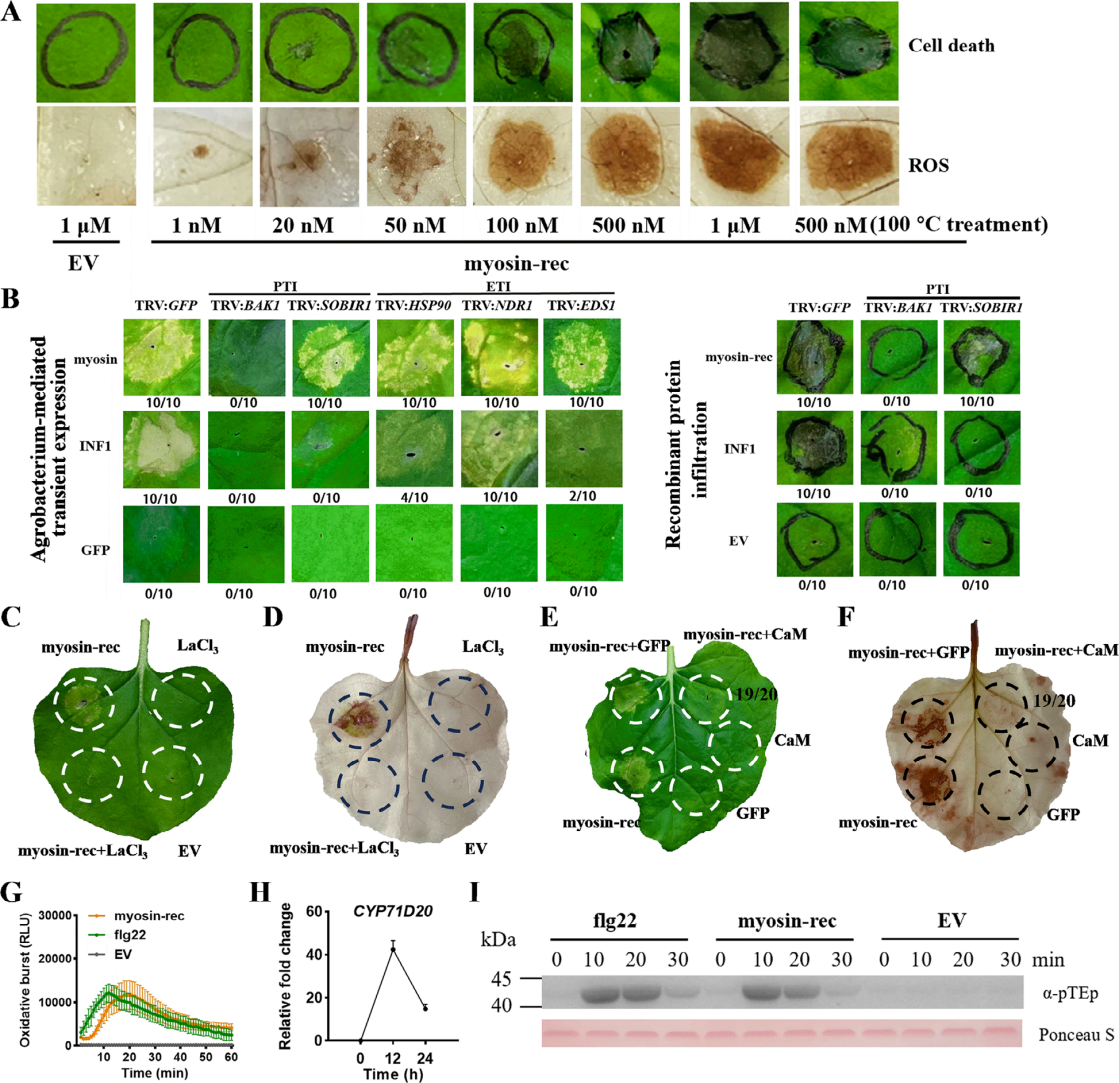
Myosin induces PTI responses in *Nicotiana benthamiana* but is not affected by heat treatment. (A) Representative leaves of *N. benthamiana* infiltrated with purified recombinant myosin‐rec protein (1 nM to 1 µM). Purified products of the empty vector (EV) served as the negative controls, and 0.5 µM myosin‐rec was treated at 100 °C for 10 min. The upper and lower panel depict detection of cell death and ROS production in *N. benthamiana*, respectively. Following DAB staining and decolorization, the intensity and area of the brown coloration corresponded to ROS levels. Photos were taken at 2 days post‐infiltration (dpi). The experiment was repeated with 20 leaves. (B) The effect of silencing the PTI and ETI related genes on myosin‐mediated cell death in *N. benthamiana*. In the left panel, *Agrobacterium* strains carrying VIGS vectors with the indicated genes were infiltrated into the leaves of *N. benthamiana*. Three weeks post‐infiltration, the *Agrobacterium* strain harboring myosin was re‐infiltrated into the same regions. Photos were taken at 4 dpi. In the right panel, 0.5 µM myosin‐rec was re‐infiltrated into the same VIGS regions, photos were taken at 2 dpi. GFP or EV an INF1 served as negative and positive controls, respectively. The numbers of fractions (e.g., 10/10) in the circle indicate the number of leaves showing cell death out of the 10 experimental leaves. (C–D) The inhibition of the calcium channel inhibitor LaCl_3_ on plant cell death (C) and ROS production (D) induced by 100 nM myosin‐rec. The inhibition effect was observed in all 20 experimental leaves of *N. benthamiana*. Photos were taken at 2 dpi. (E–F) Inhibition of myosin‐triggered cell death (E) and ROS production (F) in *N. benthamiana* leaves expressing CaM. The fraction 19/20 in the circle of representative leaves indicates reduction of myosin‐triggered cell death and ROS production in 19 leaves areas expressing CaM‐GFP relative to 20 experimental leaves, while the myosin‐induced cell death and ROS production was apparent in areas expressing GFP in all 20 experimental leaves. *N. benthamiana* leaves were infiltrated with *Agrobacterium* harboring CaM‐GFP at 24 h before 100 nM myosin‐rec infiltration into the same regions. CaM indicates the SBPH salivary effector calmodulin. Photos were taken at 2 dpi. (G) Myosin‐triggered ROS burst. Flg22 and EV were used as positive and negative controls, respectively. ROS production induced by 0.5 µM myosin‐rec was measured with a luminol‐based assay. Mean RLU [Relative Luminescence Unit (± SE)] are shown (*n* = 12). (H) Myosin‐rec treatment upregulated the expression of PTI‐associated gene *CYP71D20*. Mean fold change (+ SE, *n* = 3) was quantified by RT‐qPCR. (I) Myosin‐induced MAPK activity. *N. benthamiana* plants were treated with 0.5 µM myosin‐rec at the indicated times, and then, MAPK activity was determined by immunoblot with α‐pTEp antibody. Protein loading was indicated by Ponceau S staining for Rubisco protein.

To confirm that myosin induces plant immune responses, we infiltrated recombinant myosin protein (myosin‐rec) into the leaf of *N. benthamiana*. We found that the degree of cell death and ROS production increased with increasing concentrations of myosin‐rec, and that at concentrations of myosin‐rec greater than 50 nM, significant cell death and ROS production were triggered in the *N. benthamiana* leaves (**Figure** **2**A). Notably, myosin‐rec was capable of inducing cell death and ROS production even after heat treatment by boiling myosin‐rec for 10 min (Figure 2A). Thus, myosin‐rec functions as a thermostable protein elicitor in *N. benthamiana*.

### 2.3 Cell Death Triggered by Myosin in *N. benthamiana* Requires BAK1

To identify PTI/ETI related genes involved in myosin‐mediated cell death, we employed a VIGS assay to knockdown these genes in *N. benthamiana* (Figure S5A, Supporting Information). Expression of these genes was markedly reduced in silenced plant lines compared to in the GFP controls (Figure S5B, Supporting Information). When myosin was transiently expressed in *N. benthamiana*, a plasmolysis assay revealed that it was primarily localized to the apoplast (Figure S2D, Supporting Information), and it triggered cell death in VIGS‐GFP control plants as expected; however, they failed to induce cell death in BAK1‐silenced plants (Figure 2B). Interestingly, SOBIR1 and several NLR protein‐related signaling components involved in ETI, including HSP90, NDR1, and EDS1, were not essential for myosin‐induced cell death. Moreover, the infiltration of myosin‐rec into *N. benthamiana* leaves triggered cell death, a process that also required BAK1 (Figure 2B). These results indicate that BAK1 is essential for myosin‐triggered cell death in *N. benthamiana*.

### 2.4 Myosin Triggered PTI Responses in *N. benthamiana*

The calcium channel inhibitor lanthanum chloride (LaCl_3_) was shown to block cell death and ROS production induced by myosin‐rec in *N. benthamiana*, indicating that the myosin‐mediated immune responses rely on the activation of calcium signaling pathways (Figure 2C,D). Moreover, myosin‐rec triggered a burst of ROS in *N. benthamiana*, with the PAMP flg22 serving as a positive control (Figure 2G). Additionally, key PTI marker genes, including *CYP71D20*, *WRKY7*, *WRKY8*, and *Acre31*,^[^ ^11^ ^]^ were significantly upregulated in response to myosin‐rec treatment (Figure 2H and Figure S6A, Supporting Information). Phosphorylation of mitogen‐activated protein kinases (MAPKs) was also observed following myosin‐rec treatment (Figure 2I). Consistent with these results, we observed similar myosin‐induced PTI responses, including the activation of calcium signaling pathways and the upregulation of key PTI marker genes, using the transient expression system (Figure S7, Supporting Information). Collectively, myosin triggers multiple PTI responses in *N. benthamiana*, including the activation of calcium signaling pathways, ROS bursts, and MAPK phosphorylation, suggesting that myosin is likely to function as a HAMP and activate a PTI pathway.

### 2.5 Myosin‐Triggered Immune Responses Are Suppressed by the SBPH Effector Calmodulin

Given that SBPH can effectively infest host plants, we hypothesized that the defense responses activated by myosin might be inhibited by SBPH effectors. To test this, we expressed the conserved salivary effector calmodulin (CaM) from SBPH in *N. benthamiana*.^[^ ^27^ ^]^ CaM can bind cytosolic Ca^2+^ and disrupt calcium signaling pathways, which are activated early in the PTI process.^[^ ^27^, ^28^ ^]^ Our results demonstrated that CaM‐GFP expression significantly reduced myosin‐rec‐induced cell death and ROS production, whereas control expression of GFP did not have this effect (Figure 2E,F). Thus, the immune responses triggered by myosin can be neutralized by the SBPH effector CaM.

### 2.6 A 41‐Amino‐Acid Peptide within Myosin (MP41) Acts as a Minimal Immunogenic Epitope and Induces PTI Responses in *N. benthamiana*

**Figure 3:**
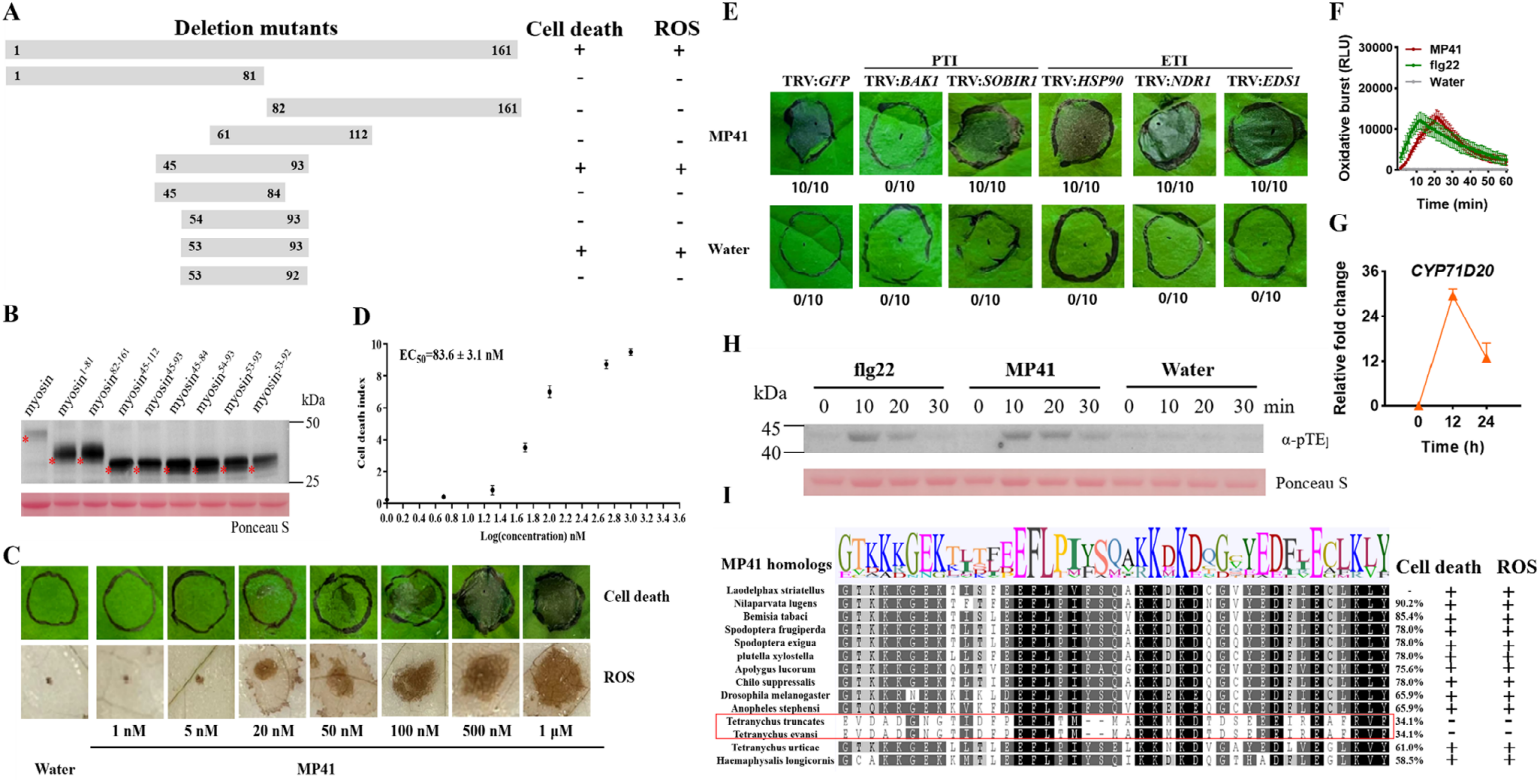
The minimal immunogenic epitope MP41 induces PTI responses in *Nicotiana benthamiana*. (A) Cell death and ROS burst symptoms in *N. benthamiana* leaves expressing myosin deletion mutants. The symbols + and – represent the presence and absence of cell death and ROS burst symptoms, respectively. (B) Western blot analysis of the amount of indicated peptides transiently expressed in *N. benthamiana*. Equal loading of plant samples was indicated by Ponceau S staining of the Rubisco protein. The red asterisks (*) indicate the expressed peptides. (C) Representative *N. benthamiana* leaves infiltrated with synthetic MP41 (1 nM to 1 µM). The experiment was repeated with 20 leaves. Photos were taken at 2 dpi. (D) Determination of MP41 EC_50_ values in cell death‐inducing activity, based on cell death index (Figure S16). Values are means ± SE, *n* = 5. (E) Silencing *bak1* inhibited MP41‐mediated cell death in *N. benthamiana*. *Agrobacterium* strains carrying VIGS vectors with the indicated PTI and ETI related genes were infiltrated into the leaves of *N. benthamiana*. Three weeks post‐infiltration, 0.5 µM MP41 was re‐infiltrated into the same regions, photos were taken at 2 dpi. Water served as negative control. The numbers of fractions (e.g., 10/10) in the circle indicate the number of leaves showing cell death out of the 10 experimental leaves. (F) MP41‐triggered ROS burst. Flg22 and water were used as positive and negative controls, respectively. ROS production was measured with a luminol‐based assay. Mean RLU [Relative Luminescence Unit (± SE, *n* = 12)] are shown. (G) MP41 upregulated the expression of the PTI‐associated gene *CYP71D20*. Mean fold change (+ SE, *n* = 3) was quantified by RT‐qPCR. (H) MP41‐induced MAPK activity. *N. benthamiana* plants were treated with 0.5 µM MP41 at the indicated times, and then, MAPK activity was determined by immunoblot with α‐pTEp antibody. Protein loading was indicated by Ponceau S staining for Rubisco protein. (I) Sequence alignment of MP41 homologs from various arthropod species. Black shading indicates conserved amino acids within MP41s. The percentage in the figure represents the identity of the MP41 homologs with MP41. The peptides of MP41 homologs from different arthropod species were synthesized and tested their capacity to induce cell death and ROS production in *N. benthamiana*. The MP41 variants from *T. truncatus* and *T. evansi* did not induce cell death or ROS production, whereas MP41 variants from the other 12 species tested were able to do so.

To identify the minimal immunogenic epitope within myosin responsible for elicitor activity, we generated truncated mutant variants of myosin and assessed their ability to induce cell death and ROS production in *N. benthamiana* using agroinfiltration (**Figure** **3**A). Initially, because myosin doesn't have any conserved domains, we divided it into two equal segments (1‐81 and 82–161) and found that neither could trigger cell death and ROS production. We therefore speculated that the immunogenic segment must span these two segments. To test this, we constructed various truncated peptides targeting the middle region, ultimately narrowing our focus to the immunogenic epitope spanning amino acids 53–93 (MP41; Figure 3A). Neither the 54–93 nor the 53–92 segment induced cell death or ROS production. Additionally, western blot analysis showed the accumulation of the variants, and no significant differences in protein abundance via expressing the MP41, 54–93, or 53–92 segments (Figure 3B), suggesting that the observed cell death and ROS production are independent of the expression levels of these peptides. Thus, MP41 is the minimal immunogenic epitope responsible for eliciting immune responses.

To further confirm the HAMP function of MP41, we synthesized MP41 and tested its capacity to induce cell death and ROS production in *N. benthamiana* at concentrations ranging from 1 nM to 1 µM. The degree of cell death and ROS production increased with increasing concentrations of the synthetic peptides (Figure 3C). The half‐maximal effective concentration (EC_50_) value was calculated to be 83.6 nM (Figure 3D). Furthermore, MP41‐triggered cell death in *N. benthamiana* requires BAK1 (Figure 3E). MP41 also triggers multiple PTI responses in *N. benthamiana*, including ROS bursts (Figure 3F), upregulation of PTI marker genes (Figure 3G and Figure S6B, Supporting Information), and phosphorylation of MAPKs (Figure 3H). Thus, MP41 induces a range of PTI responses in *N. benthamiana*, consistent with the effects observed with myosin. To examine the distinctiveness of plant responses to MP41, we directly infiltrated the synthetic peptides into expanded leaves of various plants. MP41 induced localized cell death in pepper (*Capsicum annuum*), eggplant (*Solanum melongena*), cotton (*Gossypium hirsutum*), wheat (*Triticum aestivum*), and rice, but not in tomato (*Solanum lycopersicum*), maize (*Zea mays*), or barley (*Hordeum vulgare*) (Figure S8, Supporting Information). Collectively, MP41 can induce cell death in at least five different plant species.

### 2.7 MP41 Homologs from Diverse Arthropod Species Induces Cell Death and ROS Production

The finding that MP41‐induced cell death occurs in various plant species suggests an ancient interaction between myosin and plants. Moreover, myosin is widely found in arthropods. We analyzed the phylogeny of this protein, and found that myosin homologs are grouped into distinct clusters that cover the broader arthropod classifications of Arachnida (including *Tetranychus evansi*, *Tetranychus urticae*, *Tetranychus truncatus*, and *Haemaphysalis longicornis*) and Insecta (including *Nilaparvata lugens, Bemisia tabaci*, *Spodoptera frugiperda*, *Spodoptera exigua*, *Plutella xylostella*, *Apolygus lucorum*, *Chilo suppressalis*, *Drosophila melanogaster*, and *Anopheles stephensi*) (Figure 3I). Protein sequence alignments indicate that these homologous proteins share 30–91% sequence identities with SBPH myosin. These results suggest that myosin is an ancient protein conserved across the arthropods.

Given the conservation of myosin sequences, we hypothesized that plants have a wide ability to detect myosin homologs. To test this hypothesis, we cloned MP41s within myosin homologs across a diverse range of feeding guilds. This included *N. lugens* and *B. tabaci*, hemipteran pests that feed on phloem sap; lepidopteran pests such as *S. frugiperda*, *S. exigua*, *P. xylostella*, *A. lucorum*, *C. suppressalis*, which chew on plant tissues; *D. melanogaster*, a dipteran insect that consumes spoiled fruits; three species of tetranychus spider mites—*T. evansi*, *T. urticae* and *T. truncatus*; *A. stephensi*, a dipteran insect, and *H. longicornis*, a tick, both feeding on mammalian blood. Our results showed that, despite their different feeding modes, both piercing‐sucking and chewing herbivorous arthropods had MP41 homologs that triggered cell death and ROS accumulation (Figure 3I). Interestingly, MP41 homologs from sanguivorous arthropods also elicited defense responses (Figure 3I), even though these MP41s may never directly contact live plant cells. In contrast, MP41 homologs from *T. evansi* and *T. truncatus* did not trigger defense responses, possibly due to significant divergence in their amino acid sequences (Figure 3I). Protein sequence alignments of these MP41s indicate that the homologous MP41s that can induce immune responses share sequence identities of 61–90% with SBPH MP41, while the MP41s of *T. evansi* and *T. truncates* share only 34% sequence identity with that of SBPH MP41 (Figure 3I). These findings suggest that some MP41 homologs are recognized by plants as potential HAMPs, with recognition extending beyond herbivorous to include non‐phytophagous arthropods.

### 2.8 Myosin/MP41 Induces PTI Responses in Rice

**Figure 4:**
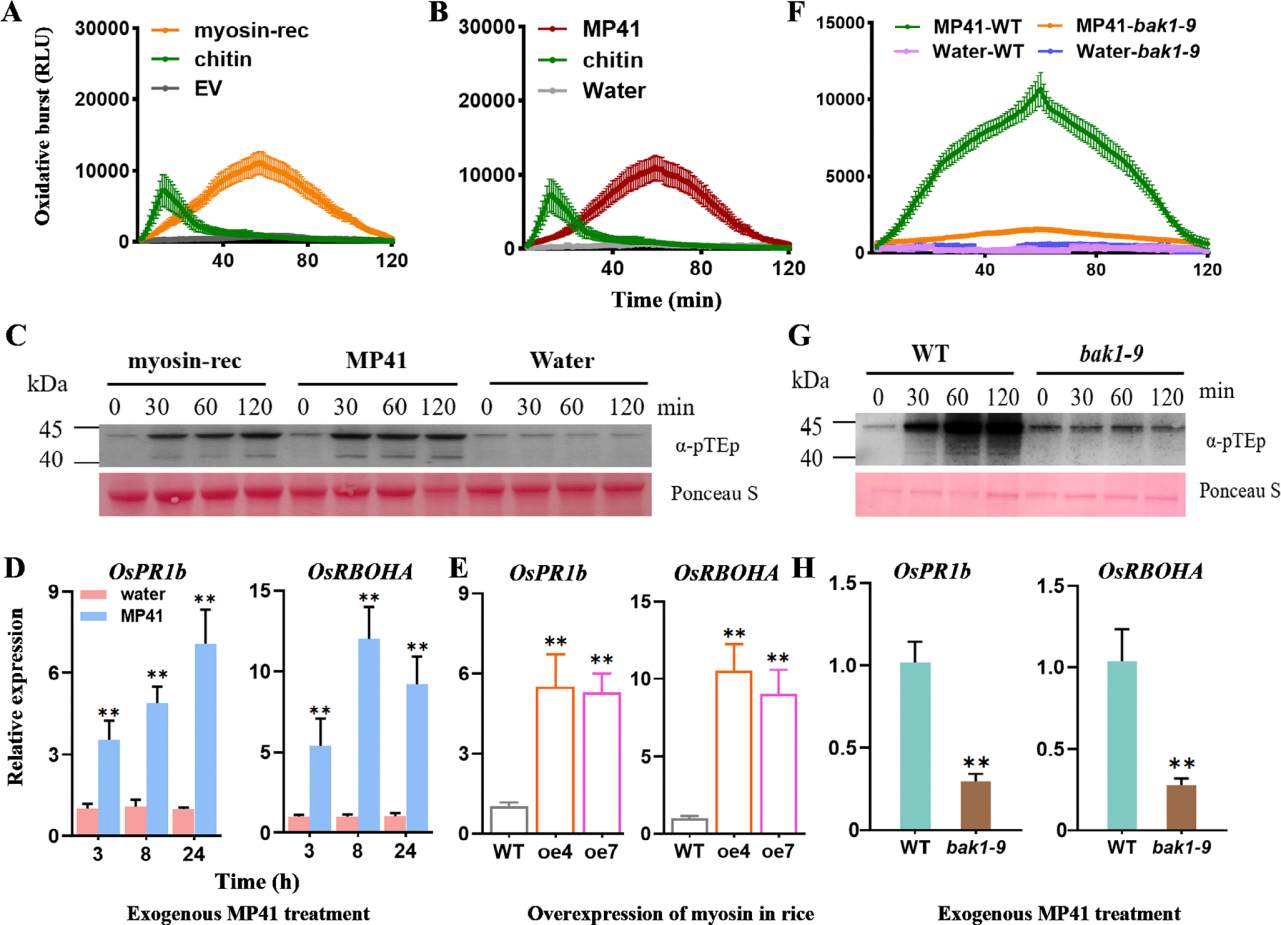
MP41 induces multiple PTI responses in rice. (A and B) Rice ROS production dynamics induced by 0.5 µM myosin‐rec (A) or 0.5 µM synthetic MP41 (B). Chitin and EV/water served as positive and negative controls, respectively. ROS production was measured with a luminol‐based assay. Mean RLU [Relative Luminescence Unit (± SE, *n* = 12)] is shown. (C) MAP kinase activity in rice stems 0, 30, 60, and 120 min after application of 0.5 µM MP41 or water to fresh wounds. MAPK activity was determined by immunoblot with α‐pTEp antibody. Protein loading was indicated by Ponceau S staining for Rubisco protein. (D and E) MP41 treatment (D) and overexpression of myosin (E) upregulated the expression of PTI‐associated genes, *OsPR1b* and *OsRBOHA*, in rice. Mean fold change (+ SE, *n* = 3) was quantified by RT‐qPCR. (F) ROS production dynamics in WT and *bak1‐9* mutant rice leaf discs treated with 0.5 µM MP41 or water. (G) MAPK activity in WT and *bak1‐9* rice stems 0, 30, 60, and 120 min after application of 0.5 µM MP41 to fresh wounds. (H) Mean expression levels (+ SE, *n* = 3) of *OsPR1b* and *OsRBOHA* in WT and *bak1‐9* rice stems treated with 0.5 µM MP41 to fresh wounds. Asterisks indicate significant difference between treatments (**p < 0.01; Student's *t*‐tests).

Given that myosin and MP41 induce PTI responses in *N. benthamiana*, we sought to investigate whether they can also induce PTI responses in rice. The results show that both myosin‐rec and MP41 treatment can trigger ROS bursts (**Figure** **4**A,B) and phosphorylation of MAPKs (Figure 4C) in rice. Furthermore, the rice PTI marker genes *PATHOGENESIS‐RELATED 1b* (*OsPR1b*) and *RESPIRATORY BURST OXIDASE HOMOLOG A* (*OsRBOHA*)^[^ ^29^ ^]^ were significantly upregulated in response to MP41 treatment (Figure 4D). To further understand how overexpression of myosin in rice influences these PTI marker genes, we generated two independent transgenic T_2_ homozygous rice lines (oe4 and oe7) that constitutively express *myosin*. Myosin expression was confirmed by measuring its transcript levels and protein levels (Figure S9A, Supporting Information). Eight hours after SBPH feeding, the transcript levels of *OsPR1b* and *OsRBOHA* were significantly higher in the myosin‐expressing lines compared to (wild‐type) WT plants (Figure 4E). Furthermore, we examined whether OsBAK1 influences MP41‐mediated PTI responses using the previously reported *bak1* rice mutant, *bak1‐9*.^[^ ^29^ ^]^ The MP41‐induced ROS burst peak in the *bak1‐9* mutant was significantly lower than in WT leaves (Figure 4F). Moreover, MP41‐induced MAPK phosphorylation was suppressed in *bak1‐9* plants compared to WT rice (Figure 4G). Eight hours after MP41 treatment, the transcript levels of *OsPR1b* and *OsRBOHA* were significantly lower in *bak1‐9* plants compared to WT plants (Figure 4H). Thus, myosin and MP41 induce PTI responses in rice, and this process requires OsBAK1.

### 2.9 Myosin Induces Production of JA, JA‐Ile, and H2O2 in Rice

**Figure 5:**
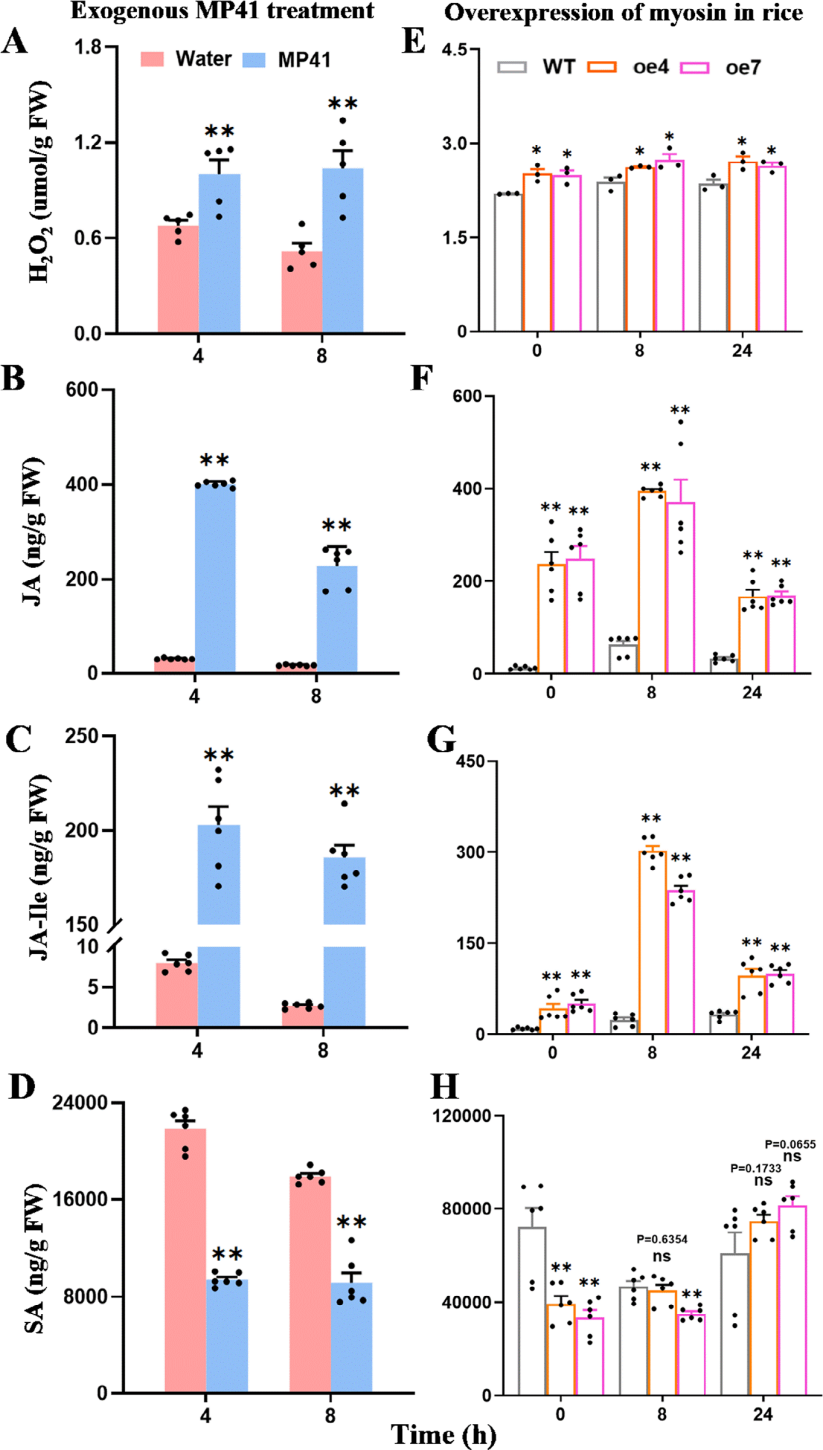
Myosin/MP41 induces the accumulation of JA, JA‐Ile, and H_2_O_2_ in rice. (A–D) Mean levels (+ SE, *n* = 5–6) of H_2_O_2_ (A), JA (B), JA‐Ile (C), and SA (D) in rice stems 4 and 8 h after application of 0.5 µM synthetic MP41 or water to fresh wounds. (E–H) Mean levels (+ SE, *n* = 5–6) of H_2_O_2_ (E), JA (F), JA‐Ile (G), and SA (H) in myosin‐expressing rice lines (oe4 and oe7) compared to WT rice plants. Each rice plant was infested with 20 newly emerged SBPH female adults for 0, 8, or 24 h. Asterisks indicate significant difference between treatments (*p < 0.05; **p < 0.01; Student's *t*‐tests).

JA signaling pathways play a central role in regulating the resistance of rice to planthoppers and striped stem borer (SSB).^[^ ^30^, ^31^ ^]^ Exogenous application of MP41 to fresh wounds led to significant increases in H_2_O_2_, JA, and JA‐Ile accumulation in rice, with increases of ≈1‐fold, 12‐fold, and 24 to 67‐fold, respectively, after 4 and 8 h of treatment (**Figure** **5**A–C). Furthermore, we tested whether overexpression of myosin in rice also influences the defense‐related signaling. Compared to WT plants, the myosin‐expressing lines exhibited a significant (≈0.2‐fold) increase in H_2_O_2_ accumulation, regardless of SBPH infestation (Figure 5E). The levels of JA rose by ≈19‐fold in myosin‐expressing lines in uninfested plants, and about 5‐fold following SBPH attack (Figure 5F). Additionally, JA‐Ile levels increased by roughly 6‐fold in the absence of SBPH and between 3‐ and 12‐fold in the presence of SBPH in the myosin‐expressing lines (Figure 5G). However, rice plants expressing myosin exhibit reduced plant height compared to WT plant (Figure S9B–D, Supporting Information), suggesting the trade‐off between growth and defense.

Compared to water control, SA levels in rice plants treated with MP41 decreased significantly, to ≈0.5‐fold (Figure 5D). In the absence of SBPH infection, SA levels in myosin‐expressing lines were significantly lower than in WT plants, decreasing to ≈0.5‐fold. Eight hours post‐SBPH infection, the oe4 line still exhibited significantly lower SA levels, while oe7 also showed lower levels, although the difference with WT was not statistically significant. However, 24 h following SBPH infection, SA levels in both oe4 and oe7 exceeded those of WT plants slightly (Figure 5H).

### 2.10 Myosin Induces Rice Defense Response against SBPH

We hypothesized that these dramatic increases in JA and JA‐Ile accumulation would further affect the performance of SBPH (direct defense) as well as the behavioral response of *Anagrus nilaparvatae*, a parasitoid of rice planthoppers (indirect defense).

**Figure 6:**
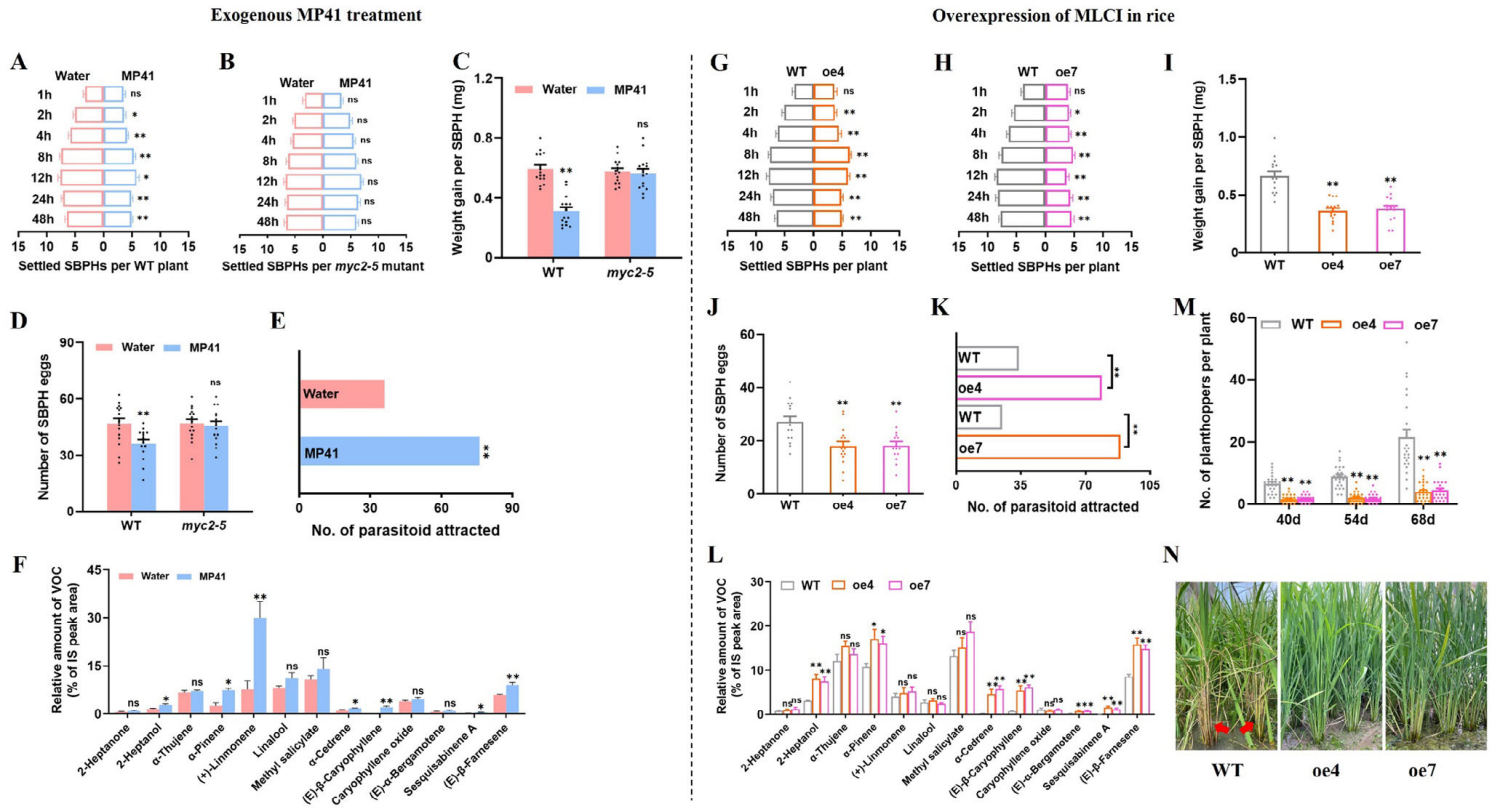
Myosin/MP41 enhances rice's direct and indirect defenses against SBPH. (A–B) SBPH host preference in choice tests on WT (A) and *myc2‐5* mutant (B) plants. Mean number (+ SE, *n* = 10) of nymphs per plant for pairs of plants treated with 0.5 µM MP41 or water to fresh wounds. Fifteen fourth‐instar nymphs were released between two rice plants, and the number of nymphs on each rice was recorded at 1, 2, 4, 8, 12, 24, or 48 h after releasing. (C) Mean weight gain (+ SE, *n* = 15) per SBPH nymph fed on WT or *myc2‐5* rice plants treated with 0.5 µM MP41 or water to fresh wounds for 7 days. (D) Mean number of eggs (+ SE, *n* = 15) laid by a SBPH female adult on WT or *myc2‐5* rice plants treated with 0.5 µM MP41 or water to fresh wounds. (E) Number of newly emerged *Anagrus nilaparvatae* female adults attracted to volatiles emitted from MP41‐treated and water‐treated rice plants. (F) Mean amount (% of IS peak area, + SE, *n* = 6) of volatile organic compounds (VOC) emitted from MP41‐treated and water‐treated rice plants. (G–H) SBPH host preference in choice tests on myosin‐expressing lines or WT plants. (I) Mean weight gain (+ SE, *n* = 15) per SBPH nymph fed on myosin‐expressing lines or WT plants for 7 days. (J) Mean Number of eggs (+ SE, *n* = 15) laid by a SBPH female adult on myosin‐expressing lines or WT plants. (K) Number of newly emerged *A. nilaparvatae* female adults attracted to volatiles emitted from myosin‐expressing lines or WT plants. (L) Mean amount (% of IS peak area, +SE, n = 6) of VOC emitted from myosin‐expressing lines and WT plants. (M–N) The performance of planthoppers on myosin‐expressing lines and WT plants in the field. (M) Mean number (+ SE, *n* = 24) of planthoppers per plant on myosin‐expressing lines and WT plants. (N) Damage phenotypes of WT plants and myosin‐expressing lines by planthoppers in the field. Pictures were taken 80 d after planting. WT plants, indicated by red arrows, were severely damaged by planthoppers. Asterisks indicates significant differences between treatments (*p < 0.05; **p < 0.01; χ^2^ tests for A, B, E, G, H and K). Asterisks indicate significant difference between treatments (*p < 0.05; **p < 0.01; Student's *t*‐tests for C, D, F, I, J, L, and M).

To investigate direct defense, we assessed the effects of exogenous MP41 treatment and myosin overexpression in rice on SBPH repulsion (antixenosis) and reproduction (antibiosis) through choice and no‐choice tests. In the choice test, SBPH exhibited a strong preference for water‐treated controls plants over MP41 treatments (**Figure** **6**A). In the no‐choice test, SBPH nymphs feeding on the MP41‐treated rice plants showed reduced weight gain and egg production compared to those feeding on water‐treated rice plants (Figure 6C,D). While, MP41 treatment upregulated the expression of *MYC2*, the JA‐responsive transcription factor, in rice (Figure S10, Supporting Information), and the reduction in insect performance observed in both choice and no‐choice tests diminished when these SBPHs were maintained on the JA‐deficient *myc2‐5* mutant plants treated with MP41 (Figure 6B–D). Similarly, compared to the SBPH fed on WT plants, a decrease in insect performance was observed in those fed on myosin‐expressing lines, both in choice and no‐choice tests. (Figure 6G–J). In addition, MP41 can still trigger a significant ROS burst in the *myc2‐5* plants, albeit with slightly lower intensity compared to WT plants (Figure S11, Supporting Information). This suggests that the suppression of the JA pathway has a modest impact on the MP41‐induced ROS burst.

Given that JA signaling pathways are crucial in the synthesis of herbivore‐induced plant volatiles (HIPVs), we investigated the effect of myosin on synthesis of volatiles. MP41‐treated and myosin‐expressing rice plants were more attractive to female *A. nilaparvatae* wasps compared to water‐treated or WT plants (Figure 6E,K). Volatile collection and analyses indicated that the total quantities of volatiles emitted from MP41‐treated and myosin‐expressing lines rice plants were significantly greater than from water‐treated and WT plants, respectively. Specifically, MP41 treatment emitted significantly higher amounts of volatiles, including 2‐heptanol, α‐pinene, (+)‐limonene, α‐cedrene, (E)‐β‐caryophyllene, sesquisabinene A and (E)‐β‐farnesene (Figure 6F). Similarly, compared with the WT plants, the transgenic lines emitted significantly higher levels of volatiles, including 2‐heptanol, α‐pinene, α‐cedrene, (E)‐β‐caryophyllene, (E)‐α‐bergamotene, sesquisabinene A, and (E)‐β‐farnesene (Figure 6L). Overall, these findings suggest that myosin enhances direct and indirect defense responses of rice against SBPH infestation. To further confirm these results, we conducted a field study comparing myosin‐expressing lines with WT plants. Planthoppers preferentially fed on WT plants, and we found that the number of planthoppers was significantly higher on WT plants compared to those from the myosin‐overexpressing lines (Figure 6M), leading to more severe damage of WT plants (Figure 6N).

**Figure 7:**
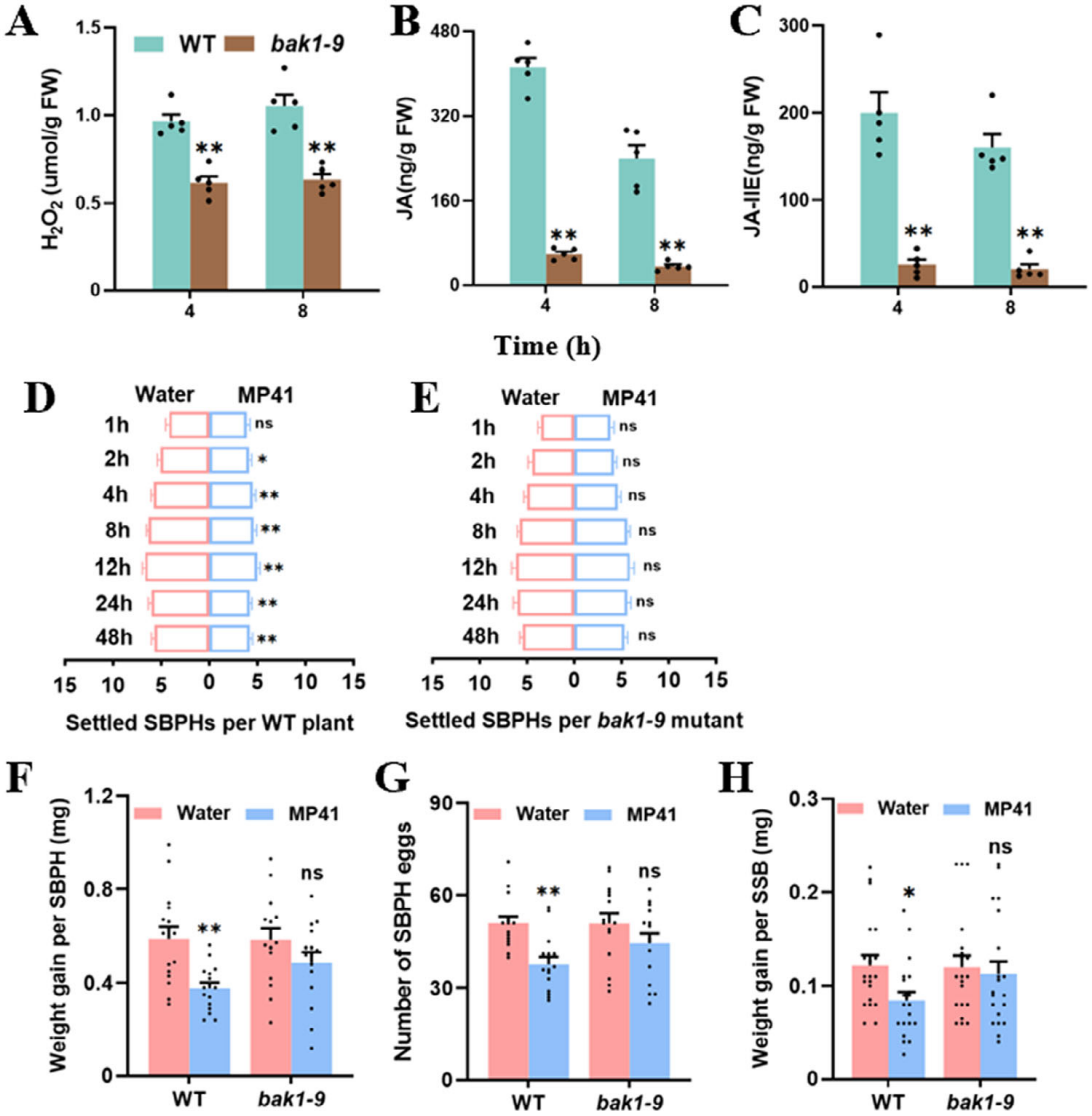
MP41‐enhanced insect resistance in rice depends on OsBAK1. (A–C) Mean levels (+ SE, *n* = 5) of H_2_O_2_ (A), JA (B), and JA‐Ile (C) in WT and *bak1‐9* mutant rice stems 4 and 8 h after application of 0.5 µM synthetic MP41 to fresh wounds. (D and E) SBPH host preference in choice tests on WT (D) and *bak1‐9* (E) plants. Mean number (+ SE, *n* = 10) of nymphs per plant for pairs of plants treated with 0.5 µM MP41 or water to fresh wounds. Fifteen fourth‐instar nymphs were released between two rice plants, and the number of nymphs on each rice was recorded at 1, 2, 4, 8, 12, 24, or 48 h after releasing. (F) Mean weight gain (+ SE, *n* = 15) per SBPH nymph fed on WT and *bak1‐9* rice plants treated with 0.5 µM MP41 or water to fresh wounds for 7 days. (G) Mean number of eggs (+ SE, *n* = 15) laid by a SBPH female adult on WT and *bak1‐9* rice plants treated with 0.5 µM MP41 or water to fresh wounds. (H) Mean weight gain (+ SE, *n* = 20) per striped stem borer (SSB) on WT and *bak1‐9* rice plants treated with 0.5 µM MP41 or water to fresh wounds 7 days after infestation. Asterisks indicate significant differences between treatments (*p < 0.05; **p < 0.01; Student's *t*‐tests for A–C and F–H). Asterisks indicate significant differences between treatments (*p < 0.05; **p < 0.01; χ^2^ tests for D and E).

Furthermore, we investigated whether MP41‐induced defense‐related signaling and insect resistance are dependent on OsBAK1. Eight hours after MP41 treatment, the production of H_2_O_2_ (**Figure** **7**A), JA (Figure 7B), and JA‐Ile (Figure 7C) in *bak1‐9* was significantly lower compared to WT. Moreover, In the choice test, SBPH exhibited a strong preference for water‐treated controls plants over MP41 treatments (Figure 6A). In the no‐choice test, SBPH nymphs feeding on the MP41‐treated rice plants showed reduced weight gain and egg production compared to those feeding on water‐treated rice plants (Figure 6C,D).MP41 treatment on WT rice enhanced resistance to SBPH and SSB. Compared to water‐treated WT plants, MP41‐treated WT plants repelled SBPH (Figure 7D) and reduced both SBPH weight gain and egg production (Figure 7F,G), as well as decreased SSB weight gain (Figure 7H). However, the reduction in above insect performance was diminished when SBPH (Figure 7E–G) or SSB (Figure 7H) were maintained on MP41‐treated *bak1‐9* plants. Therefore, MP41‐enhanced insect resistance is dependent on OsBAK1.

### 2.11 Myosin Positively Modulates Rice Defense against Rice Blast and RSV

**Figure 8:**
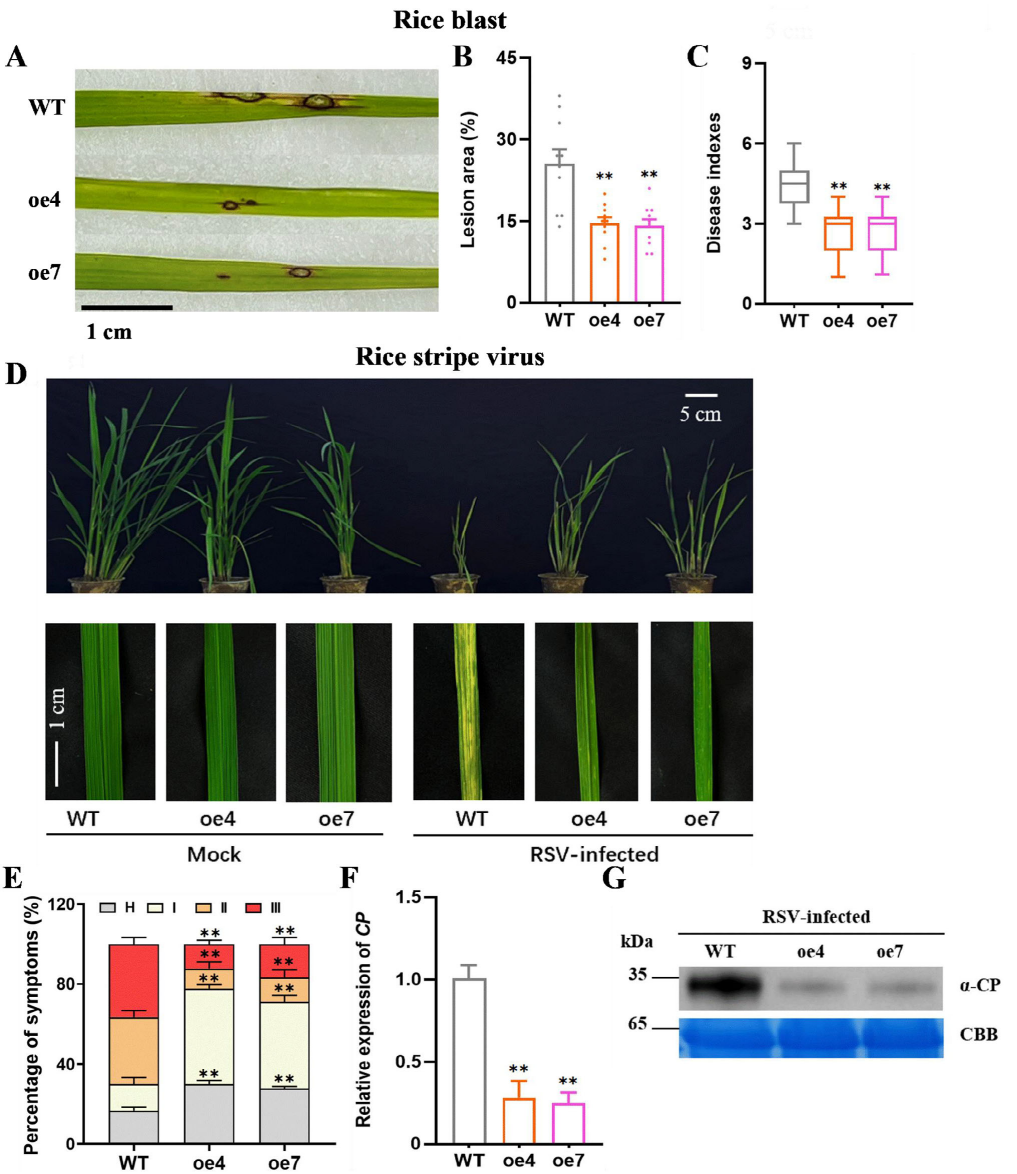
Myosin confers rice resistance to rice blast and RSV. (A) Rice disease symptoms caused by rice blast Guy11 in myosin‐expressing lines and WT rice plants. Bar = 1 cm. Photographs were taken at 7 dpi. The experiment was repeated on 30 leaves. (B–C) Mean lesion areas (+ SE, *n* = 8; B) and disease index (± SE, *n* = 30; C) in myosin‐expressing lines and WT plants, measured 7 days after rice blast infection. (D) Viral symptoms in myosin‐expressing lines and WT rice plants following RSV infection. Photographs were taken at 30 dpi. Bars = 5 cm or 1 cm. The experiment was repeated with 30 rice plants. (E) Disease incidence of RSV. Different grades of disease symptoms (+ SE, *n* = 30) in myosin‐expressing lines and WT rice plants. (F–G) Mean relative mRNA levels (+ SE, *n* = 3; F) and protein abundance (G) of RSV CP in RSV‐infected rice plants, determined with RT‐qPCR and western blotting at 30 dpi. CBB staining was shown as the protein loading control. Asterisks indicate significant difference on myosin‐expressing lines compared with WT plants (*p < 0.05; **p < 0.01; Student's *t*‐tests).

Given the increased accumulation of JA, JA‐Ile, and H_2_O_2_ in myosin‐expressing lines, we investigated the effect of myosin on infestation with fungal, bacterial, or viral pathogens. myosin‐expressing lines exhibited enhanced resistance to the rice blast fungus, *Magnaporthe oryzae*, compared to WT plants (**Figure** **8**A), and lesion areas (Figure 8B) and disease indexes (Figure 8C) in the leaves of transgenic lines were significantly lower than those in WT plants following *M. oryzae* infection. In a further experiment, myosin‐expressing lines and WT plants were inoculated with RSV. WT plants exhibited typical yellow stripe symptoms following infection, however, myosin‐expressing lines displayed milder and discontinuous yellow stripes (Figure 8D). Moreover, the transgenic lines showed less severe stunting and lower percentages of severe disease symptoms compared to WT plants at approximately 30 days post‐inoculation (Figure 8E and Figure S12, Supporting Informatio≈). RT‐qPCR and western blotting analysis showed that the transcription and protein levels of RSV coat protein (CP) were reduced in the myosin‐expressing plants compared with those in WT plants (Figure 8F,G), indicating that myosin enhanced resistance to RSV infection. Interestingly, myosin‐expressing lines and WT plants showed similar resistance to the rice bacterial blight *Xanthomonas oryzae* pv. oryzae, at 7 and 14 days after infection (Figure S13A,B, Supporting Information). Thus, myosin enhanced rice resistance to RSV and rice blast, but not rice bacterial blight.

### 2.12 MP41 Enhances the Resistances of *N. benthamiana* and *G. hirsutum* to Multiple Species of Insects

**Figure 9:**
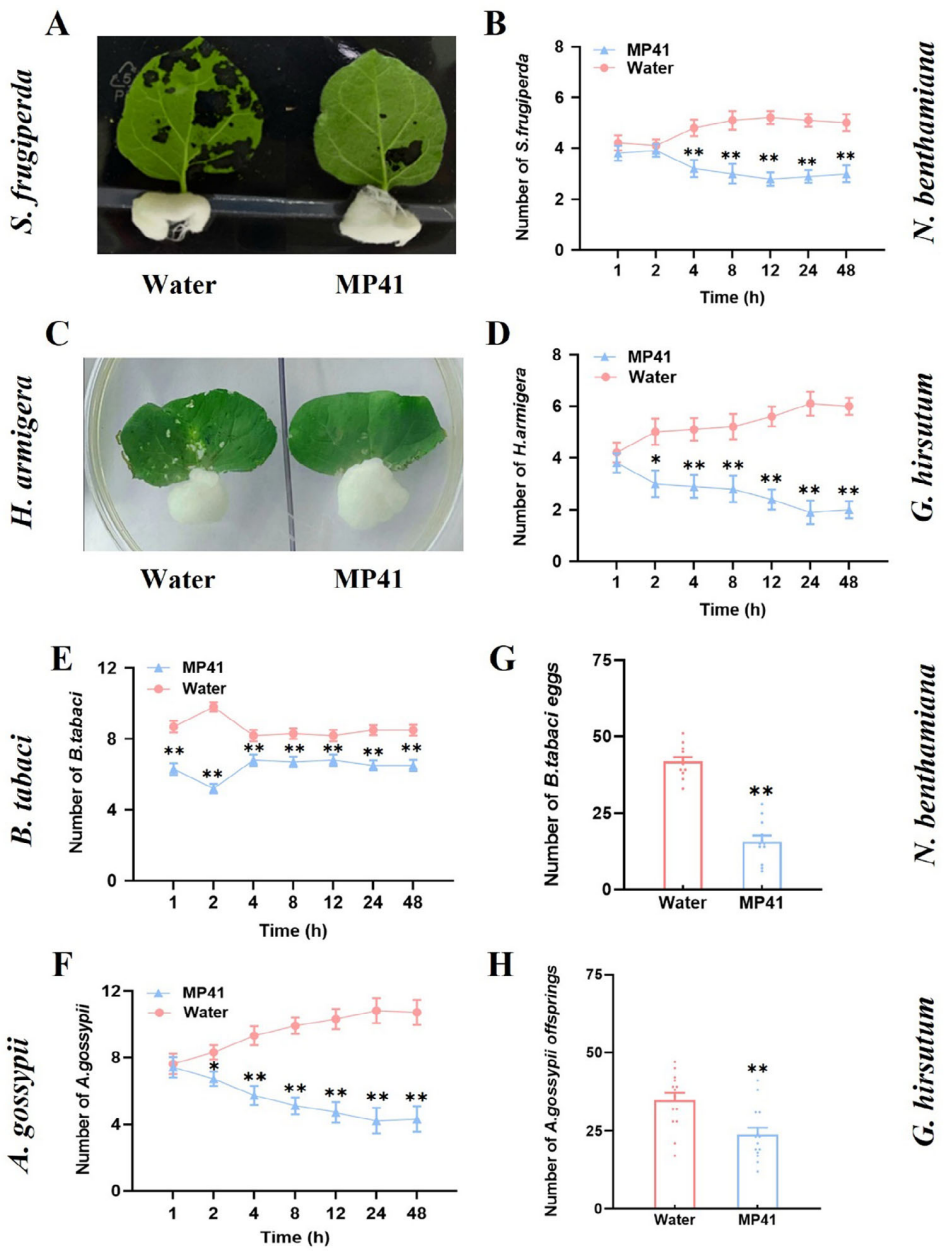
MP41 enhances the resistances of *N. benthamiana* and *G. hirsutum* to insects. (A–F) *S. frugiperda* (A and B), *H. armigera* (C and D), *B. tabaci* (E), and *A. gossypii* (F) host preference in choice tests. Mean number (+ SE, *n* = 10) of insects per plant for pairs of plants treated with 0.5 µM MP41 or water. The experiment was repeated with 10 pairs of leaves in (A) and (C). Eight second‐instar larvae of *S. frugiperda* or *H. armigera* or 15 newly emerged female adults of *B. tabaci* or *A. gossypii* were released between two treated plants, and the number of insects on each plant was recorded at 1, 2, 4, 8, 12, 24, or 48 h after releasing. (G and H) Mean number of eggs (+ SE, *n* = 15) laid by *B. tabaci* (G) and mean number of offsprings (+ SE, *n* = 15) produced by *A. gossypii* (H) on plants treated with 0.5 µM MP41 or water in non‐choice tests. Asterisks indicates significant differences between treatments (*p < 0.05; **p < 0.01; χ^2^ tests for B, D, E, and F). Asterisks indicate significant difference between treatments (**p < 0.01; Student's *t*‐tests for G and H).

The JA pathway confers protection against chewing insects and certain piercing‐sucking insects, such as aphids and whiteflies, to their host plants.^[^ ^32^, ^33^, ^34^ ^]^ MP41 induces the production of ROS and activates the expression of JA marker genes, including *NbPR3* and *NbPR4* in *N. benthamiana*, as well as *GhJAZ3* and *GhMYC2* in *G. hirsutum* (Figure 3C and Figure S14, Supporting Information),^[^ ^6^, ^35^ ^]^ so we investigated the effects of MP41 on insect performance on *N. benthamiana* and *G. hirsutum*. In choice tests, we observed lower numbers of *S. frugiperda* and *B. tabaci* on *N. benthamiana* leaves treated with MP41 compared to those treated with water (**Figure** **9**A,B,E). Similarly, *H. armigera* and *A. gossypii* were present in lower numbers on *G. hirsutum* leaves treated with MP41 compared with leaves treated with water (Figure 9C,D,G). In no‐choice tests, fewer *B. tabaci* eggs and *A. gossypii* offspring were produced on MP41‐treated leaves compared to the water controls (Figure 9F,H).

## 3 Discussion

The evolutionary arms race between plants and herbivorous insects has led to the development of numerous sophisticated defense mechanisms in plants. These defenses are often inducible, requiring plants to accurately recognize their attackers to launch an appropriate response. This recognition is facilitated by insect‐specific elicitors. Conversely, herbivorous insects face strong selective pressure to minimize the excretion of these telltale elicitors, with the expectation that only compounds they cannot avoid producing will serve as reliable elicitors. We identified the protein components of SBPH salivary sheaths, and found that the structural protein myosin, located on the sheath's surface, elicited plant immune responses. As a component of the cytoskeleton, myosin functions not only as a molecular motor but also as a scaffold that stabilizes protein complexes,^[^ ^25^ ^]^ and as part of the salivary sheath, significantly contributes to the structural integrity of the sheath. We found that myosin was essential for salivary sheath formation and likely aided planthoppers to feed on phloem sap by facilitating stylet movements and exploration of plant tissue. In a similar way, the salivary protein NIMLP from *N. lugens* not only triggers immune responses in *N. benthamiana* leaves but is also crucial for salivary sheath formation.^[^ ^7^ ^]^ The most extensively studied elicitors in chewing insects are volicitin and inceptins, which are peptide fragments derived from plant chloroplastic ATP synthase c‐subunit proteins.^[^ ^36^ ^]^ Both types of elicitors are generated in the buccal cavities of caterpillars during feeding and cannot be avoided unless insects alter their diet^[^ ^37^ ^]^ or modify their digestive enzyme activity.^[^ ^38^ ^]^ These examples indicate that elicitors play beneficial primary roles in herbivores that outweigh the costs associated with being recognized as elicitors by plants.

Most PRRs must work in conjunction with co‐receptors, which are highly conserved in land plants, to initiate downstream PTI responses. Multiple PRRs including LRR‐RLKs and LRR‐RLPs interact with the co‐receptor BAK1, while the co‐receptor SOBIR1 is essential for LRR‐RLPs signaling.^[^ ^39^ ^]^ We found that cell death induced by myosin required BAK1, but not SOBIR1, nor the key modulators of ETI signaling (which include HSP90, NDR1, and EDS1), suggesting potential recognition of myosin by LRR‐RLKs. Furthermore, myosin‐elicited immune responses exhibited characteristics common to PTI responses triggered by well‐known PAMPs, including activation of calcium pathways, ROS bursts, and activation of MAPKs. Interestingly, the triggered immune response was unaffected by boiling treatment of myosin‐rec, suggesting that the specific recognition of myosin by plants depends on the amino acid sequence rather than the secondary or 2D structure of the protein. We also identified the minimal immunogenic epitope within myosin, which consists of 41 amino acids, and we found that the synthetic peptide was sufficient to trigger PTI responses. This represents the first report of a synthetic small peptide derived from arthropod saliva enhancing plant resistance. Thus, MP41 may be recognized by a specific LRR‐RLK as a HAMP, activating PTI responses.

Most previously identified HAMPs, such as volicitin and inceptins, are predominantly found in chewing herbivores.^[^ ^36^ ^]^ Interestingly, we discovered that myosin homologs are widespread in arthropods. MP41s within myosin homologs from both piercing‐sucking and chewing herbivores, including species of the Hemiptera, Lepidoptera, Diptera, and Arachnida, elicited cell death and ROS production in *N. benthamiana*. Furthermore, myosin homologs are not only prevalent in herbivorous arthropods but are also present in blood‐sucking arthropods. Surprisingly, MP41 homologs from *A. stephensi* and *H. longicornis* also triggered immune responses in *N. benthamiana*, despite the fact that these arthropods may never interact with plant cells in nature. Given the striking similarities between plant and animal innate immunity,^[^ ^40^ ^]^ MP41 homologs in blood‐sucking arthropods may also function as molecular patterns recognized by human cells. Thus, our study provides further evidence that arthropods share common pattern molecules, despite their differing feeding behaviors and host types. However, MP41 homologs from *T. truncatus* and *T. evansi* did not induce cell death or ROS production, likely due to the differences in some amino acids. Moreover, MP41 triggered defense responses not only in Solanaceous plants, including tobacco, eggplant, and pepper, but also in Malvaceae such as cotton and in monocots such as wheat and rice. The wide taxonomic range of hosts that respond to MP41 suggests that it may be recognized by a common PRR in different plants through a conserved mechanism. Our findings support the notion that various plants can detect conserved HAMPs, thereby reducing the need for parallel, more specialized surveillance mechanisms.

The JA pathway positively regulates plant defenses against chewing insects and certain piercing‐sucking insects. In rice, the JA pathway is induced by infestations of planthoppers and stem borers, and plays a critical role in enhancing rice resistance to these pests.^[^ ^30^, ^41^ ^]^ Most previously identified salivary elicitors from planthoppers, including *NlG14*, *NlMLP*, *NlVgN*, and *LsPDI1*, activate the JA pathway.^[^ ^6^, ^7^, ^8^, ^9^ ^]^ Similarly, the overexpression of myosin in transgenic rice or the exogenous application of MP41 resulted in a dramatic increase in JA and JA‐Ile levels, thereby enhancing rice resistance to SBPH. However, the negative effects of both treatments on SBPH performance were nullified in a rice line with an impaired JA pathway, indicating that the JA pathway elicited by myosin is crucial in this process. Additionally, MP41 treatment also improved rice resistance to *C. suppressalis*, and enhanced defenses in *N. benthamiana* against *S. frugiperda* and *B. tabaci*, as well as in *G. hirsutum* against *H. armigera*, and *A. gossypii*. Thus, MP41 induces widespread resistance to insects in multiple plant species, likely through JA signaling.

The JA pathway positively regulates the biosynthesis of HIPVs in rice.^[^ ^42^, ^43^ ^]^ Consequently, the fact that myosin treatment increased the amounts of volatiles emitted from rice was probably due to myosin activation of JA pathway. Levels of eight volatile compounds were elevated in rice overexpressing myosin, and in those treated with MP41. The common volatile compounds induced by both treatments include 2‐heptanol, α‐pinene, α‐cedrene, (E)‐β‐caryophyllene, sesquisabinene A, and (E)‐β‐farnesene, all of which were also induced by *N. lugens* infestation of rice.^[^ ^43^, ^44^ ^]^ Notably, the increases in the latter five compounds align with the treatment effects of the previously identified elicitor NlVgN from *N. lugen*s on rice.^[^ ^9^ ^]^ Of the six common volatile compounds induced by overexpressing myosin and MP41 treatment, α‐pinene and (E)‐β‐caryophyllene are known to attract *A. nilaparvatae*.^[^ ^43^, ^44^ ^]^ However, 2‐heptanol, α‐cedrene, sesquisabinene A, and (E)‐β‐farnesene have not been reported whether they have the ability to attract *A. nilaparvatae*. (E)‐β‐caryophyllene and (E)‐β‐farnesene can attract other parasitoids in different plant systems, such as *Cotesia marginiventris* in maize, *Peristenus spretus* and *Aphidius gifuensis* in cotton, and *Eupeodes corollae* in broad bean.^[^ ^46^, ^47^, ^48^, ^49^ ^]^ Additionally, 2‐heptanol has been shown to repel *N. lugens*.^[^ ^43^ ^]^ Therefore, the increased attractiveness to *A. nilaparvatae* is likely due to elevated levels of α‐pinene and (E)‐β‐caryophyllene and possibly other volatiles. Myosin is not only an elicitor of direct defenses, but is also involved in volatile‐mediated indirect defense that results in the attraction of parasitoids.

Nevertheless, the feeding of planthopper nymphs, males, and newly emerged brachypterous female adults – during which no eggs are laid – does not affect the accumulation of JA and JA‐Ile in rice.^[^ ^9^, ^30^ ^]^ Moreover, SBPH feeds most rice cultivars successfully, believed to stem from the evolved ability of SBPH to evade or counteract the rice defense responses triggered by myosin and other elicitors. Secretion of salivary effectors into host plant cells is an example of such a strategy employed by herbivores to suppress elicitor‐triggered immunity, thereby facilitating their feeding.^[^ ^21^ ^]^ Ca^2+^ serves as a well‐known conserved secondary signaling molecule in eukaryotes. In rice, an influx of Ca^2+^, which is a common initial reaction in PTI, is involved in the early defense response to planthopper feeding. Furthermore, the cell death and ROS production induced by myosin were dependent on the Ca^2+^ influx. Our previous research identified SBPH salivary effector CaM, which is also conserved across arthropods, and which can bind cytoplasmic Ca^2+^ and suppress immune responses triggered by the elicitor LsPDI.^[^ ^27^ ^]^ Here, we also found that CaM inhibited the cell death and ROS production triggered by myosin. These results suggest that some effectors may have evolved to target conserved plant defense signaling pathways and counteract elicitor‐induced defenses. This creates a potential co‐evolutionary scenario, involving broad surveillance mechanisms that detect conserved elicitors, as well as conserved and specialized effector repertoires to evade such detection.

Rice crops are frequently exposed to infections from various viral, fungal, and bacterial pathogens. The JA pathway also plays a critical role in rice defense against certain major pathogens, such as RSV and rice blast.^[^ ^50^, ^51^ ^]^ The overexpression of myosin in rice enhances its resistance both to RSV and to rice blast infection. Therefore, myosin induces rice broad‐spectrum resistance to pathogens, likely through JA signaling. However, we found no difference in resistance to rice bacterial blight between myosin‐expressing lines and WT plants, likely because the SA pathway also plays an important role in rice defense against this disease,^[^ ^52^ ^]^ and because SA levels in myosin‐expressing rice plants were lower than in the WT plants.

**Figure 10:**
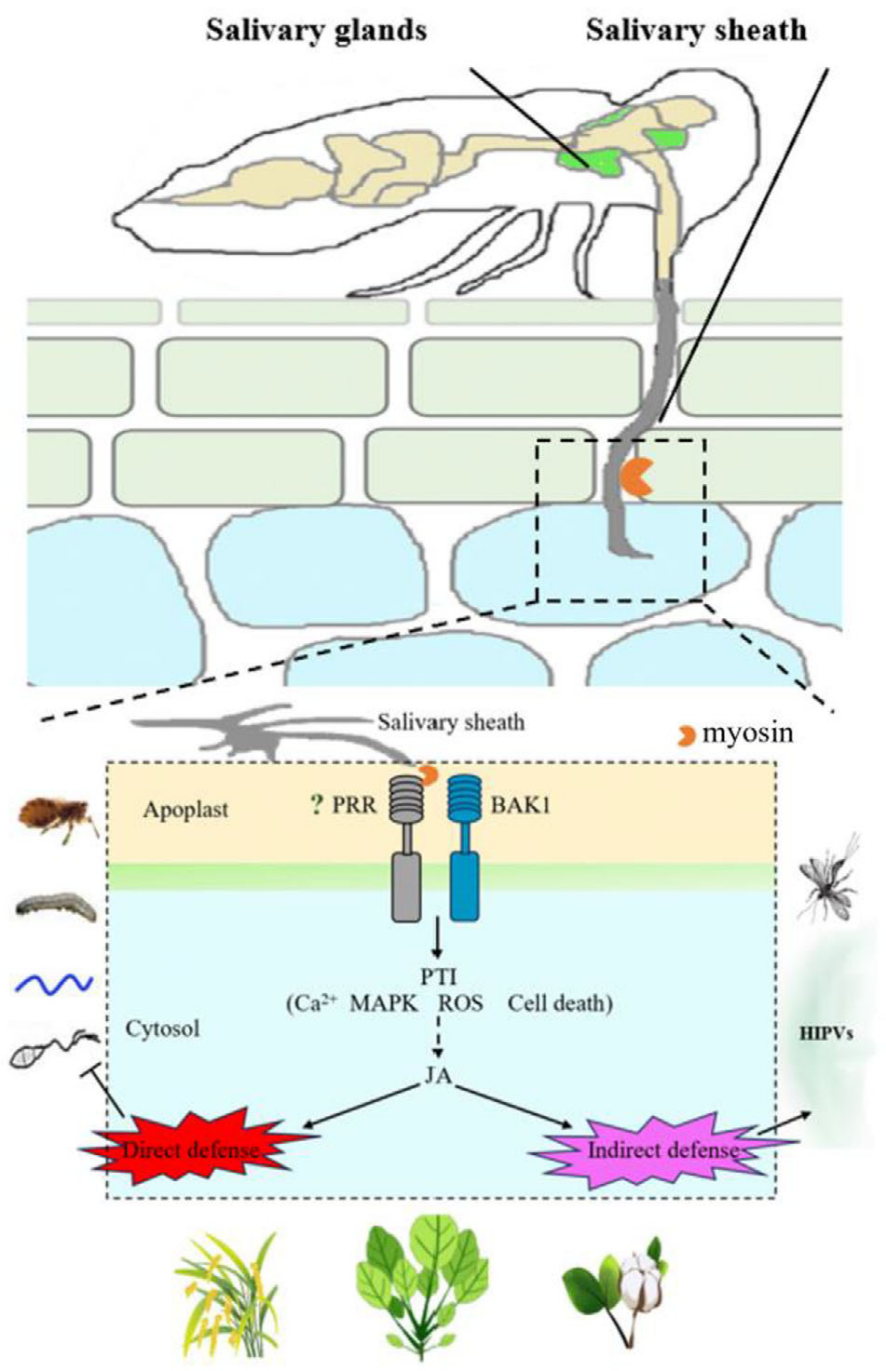
A model illustrating the role of salivary sheath protein myosin in mediating the activation of plant defense responses. The salivary sheath protein myosin from SBPH was critical for the formation of the salivary sheath and feeding. However, myosin functioned as a HAMP and triggered plant BAK1‐mediated PTI responses, which include the activation of calcium signaling pathways, MAPK phosphorylation, ROS bursts, and cell death, thereby triggering JA pathway. The minimal immunogenic peptide, MP41, was characterized. The resulting defenses not only diminished planthopper performance directly but also induced volatile emissions in rice, attracting a common parasitoid. Moreover, myosin enhanced the rice's broad‐spectrum resistance to various insects and pathogens. Additionally, myosin proteins were conserved across arthropods and induced defense response in multiple plant species.

Overall, our results provide important mechanistic insights into how a structural protein in salivary sheath affects insect feeding and how the conserved HAMP induces defense responses against various insects and pathogens (**Figure** **10**). Further studies are needed to explore how its minimal immunogenic peptide MP41 is recognized by plants and how it activates PTI, particularly by investigating the corresponding PRRs. We speculate that resistance breeding could focus on mechanisms that detect conserved HAMPs, similar to the strategy used with R genes, to achieve wide resistance while minimizing opportunities for herbivore effectors to evade detection.

## 4 Experimental Section

### Insect Rearing and Plant Growth

Original colonies of SBPH were obtained from rice fields in Nanjing, China, and were maintained on rice seedlings in a climate chamber at 26 ± 1 °C under a 14 h: 10 h, light: dark photoperiod. Third‐instar SBPH nymphs were used for survival analysis. Fifth‐instar nymphs injected with dsRNA turned into newly emerged brachypterous females and were then used for honeydew and weight analyses, as well as in rice infestation experiments to assess gene expression and analysis of defense signaling molecules. The newly emerged brachypterous female adults were selected due to their high feeding capacity and the absence of egg‐laying at this stage. SBPHs carrying RSV were kindly offered by Prof. Qiufang Xu (Jiangsu Academy of Agricultural Sciences).

The rice genotypes used in the present study were cv *Nipponbare* (WT) and two independent T_2_ homozygous lines expressing myosin (oe4 and oe7) in the same background. The rice *myc2‐5* mutant and its WT XS11 were from the previous study.^[^ ^30^ ^]^ The rice *bak1‐9* mutant and its WT ZH11 were generously provided by Prof. Xiaoyang Chen (Anhui Agricultural University), which were originally from Zhao et al., 2024.^[^ ^29^, ^53^ ^]^ Pregerminated seeds were cultured in plastic bottles in a greenhouse at 28 ± 2 °C under a 14 h: 10 h, light: dark photoperiod. Each 7‐day old seedling was transferred into a 750 mL plastic pot containing potting medium (Pindstrup, Denmark). After 25 day, individual rice plants were ready for use in subsequent experiments. *N. benthamiana* was grown in a climate chamber at 23 ± 1 °C under a 16 h:8 h, light: dark photoperiod. After 4–5 weeks (five‐leaf stage), the tobacco plants were used in subsequent *A. tumefaciens*‐mediated transient expression experiments.

### Collection of SBPH Salivary Sheaths and Identification of Proteins

A total of approximately 6000 nymphs were fed on a sterile diet with 2.5% sucrose in Parafilm (Amcor, Switzerland) sachets using a two‐way tube as a feeding chamber. The diet was prepared under aseptic conditions and was filtered through a 0.22 µm syringe filter (Millipore, MA, USA). Sachets made from two layers of stretched Parafilm, each containing 100 µL artificial diet, were placed at one end of the chamber. The opposite end of the chamber was covered with nylon mesh. SBPH could feed by piercing through the inner Parafilm layer of the diet sachets, leaving behind salivary sheaths. After feeding for 48 h, the intact salivary sheaths were carefully extracted from the Parafilm membrane using forceps under a BX51 light microscope (Olympus, Japan). The protein components of the salivary sheaths were identified with LC‐MS/MS analysis by the Shanghai Applied Protein Technology Co. Ltd.

### Gene Cloning and Vector Construction

The full‐length *myosin* was obtained with RT‐PCR using total RNA isolated from SBPH, and was then cloned into the pMD19‐T vector (TaKaRa, Japan), and sequenced. The full length ORFs of candidate genes were ligated separately into the pBINPLUS‐GFP vector via a single *Bam*HI digestion site, using the In Fusion HD cloning kit (Takara, Japan) as described previously.^[^ ^30^ ^]^ The specific primers used are listed in Tables S2 and S3 (Supporting Information).

### Transient Expression of Myosin in *N. benthamiana*

The constructed pBINPLUS‐GFP vectors were introduced into the *A. tumefaciens* strain GV3101 by electroporation. The bacteria were cultured in LB liquid medium containing kanamycin and rifampicin for 24 h at 28 °C. Recombinant strains were washed three times with infiltration buffer (1 M MgCl_2_, 100 mM MES, 150 mM acetosyringone) and resuspended to an OD_600_ of 0.4. The *Agrobacterium* cell suspension was then injected into the leaves of *N. benthamiana* using a needleless syringe. GFP expression was observed 24 h later under a Zeiss LSM750 confocal laser scanning microscope (Carl Zeiss AG, Germany). At 4 dpi, leaves from agroinfiltrated plants were collected for defense‐related assays and were subsequently photographed. To detect ROS, the collected leaves were stained with 3,3‐diaminobenzidine (DAB, Sigma, USA) solution for 8 h in the dark and then destained with ethanol. INF1, a previously characterized cell death‐inducing protein from the plant pathogen *Phytophthora infestans*,^[^ ^54^ ^]^ served as a positive control, while GFP was used as a negative control.

### Secretion Prediction of Myosin

To determine whether myosin is a secreted protein, predictions of its signal peptide, the unconventionally protein secretion, and transmembrane domain were made using SignalP 6.0 (https://services.healthtech.dtu.dk/services/SignalP‐6.0/), SecretomeP‐2.0 (https://services.healthtech.dtu.dk/services/SecretomeP‐2.0/) and TMHMM 2.0 (http://www.cbs.dtu.dk/services/TMHMM/), respectively. The protein structure prediction was performed using the AlphaFold Protein Structure Database (https://alphafold.ebi.ac.uk/entry/A0A482XHJ1).

### Sequence Alignment and Phylogenetic Analysis of Myosin

Myosin homologous proteins from 13 representative arthropod species were downloaded from NCBI (https://www.ncbi.nlm.nih.gov/). For the phylogenetic analysis of myosin, multiple sequence alignments were generated using the ClustalW2 program with default parameters. Maximum‐likelihood phylogenetic dendrograms were constructed using MEGA 7 and visualized using the Interactive Tree of Life (iTOL, https://itol.embl.de/). Alignment of *MP41* homologous gene sequences was conducted using Geneious 7.

### qRT‐PCR Analysis

The relative quantification method (2^−ΔΔCt^) was used to assess expression levels. Ct values ≥35 were considered indicative of no gene expression in the sample. Each experiment included three independent biological replicates, with each replicate performed in duplicate. Primers used for qRT‐PCR were designed with Primer Premier v6.0 (Table S4, Supporting Information). *Lsactin* (accession: KC683802.1), *Nbactin* (accession: JQ256516.1), and *Osactin* (accession: KX302608.1) were used as standard controls to normalize the target genes expression in SBPH, *N. benthamiana* and rice, respectively.

### Analysis of *Myosin* Expression

Temporal and spatial expression patterns of myosin were investigated with qRT‐PCR as follows. For analysis of spatial expression patterns, salivary glands, guts, fat bodies, testes, ovaries, and carcasses from SBPH adults were dissected. For temporal expression pattern analysis, total RNA was isolated from SBPH at various developmental stages, including eggs, first to fifth instar nymphs, female adults, and male adults. Total RNA was isolated using RNAiso Plus (Takara).

### SBPH Bioassays

To evaluate the efficiency of gene silencing following dsRNA injection, *myosin* transcription in SBPHs was measured 2 to 8 days post‐injection using qRT‐PCR, with dsRNA targeting *myosin* or *GFP*.

To assess the effect of *myosin* knockdown on SBPH feeding (honeydew production and weight gain), 5 newly emerged brachypterous female adults (3 days post‐injection with *dsmyosin* or *dsGFP* or noninjected) were placed in small Parafilm sachets (2 × 2.5 cm) attached to the basal rice stem. After weighing the insects and sachets, the insects were added to the sachets, which were then secured to the plants. After 72 h, each insect and sachet were reweighed, and the changes in weight of SBPH and sachet were defined as body weight gain and honeydew weight, respectively. Weighing was performed using an electronic balance (Mettler Toledo, Switzerland). This experiment included 10 replicates per treatment and was repeated three times.

To measure survival rates of SBPH, third‐instar nymphs were injected and allowed to recover on rice seedlings for 1 day before being used in bioassays. Rice stems (one plant per pot) were enclosed in glass cylinders (2 cm diameter, 8 cm height) with releasing 20 nymphs per treatment. The number of surviving SBPH nymphs on each plant was recorded daily for 10 d. This experiment included six replicates per treatment and was repeated three times.

To evaluate the feeding preferences of SBPH on rice, a host choice test was conducted as follows: one WT Vs transgenic rice plant or one MP41‐ versus water‐treated plant was confined in a glass cylinder (8 cm diameter; 8 cm height), and 15 fourth‐instar nymphs were released. The numbers of SBPH nymphs settling on each plant were counted at 1, 2, 4, 8, 24, and 48 h post release. This experiment included ten replicates per treatment and was repeated three times.

In a no‐choice test, weight gain and fecundity tests were conducted. After weighing, 10 s‐instar nymphs were placed in glass tubes with holes and secured to basal stems of rice plants. After 7 days of feeding, the SBPHs were reweighed to assess their weight gain. For the fecundity test, one newly emerged female SBPH and two newly emerged male SBPHs were confined within a glass cylinder placed around the rice stem. Seven days later, the number of eggs laid by each female was counted under a BX51 light microscope (Olympus, Japan). This experiment included fifteen replicates per treatment and was repeated three times.

### Immunohistochemistry Staining of Salivary Sheaths

After 20 fourth‐instar SBPH nymphs had been allowed to feed on the artificial diet in Parafilm sachets for 48 h, the Parafilm membrane, together with the salivary sheaths, was removed and gently rinsed with 75% ethanol solution. The salivary sheath was then carefully picked from the Parafilm using insect needles under a light microscope, placed into PBS, and fixed in 4% paraformaldehyde for 30 min. SBPH‐infested leaf sheath samples were cut into 2 cm segments and fixed in 4% paraformaldehyde solution. After fixation, the samples were dehydrated with ethanol, and were then embedded in paraffin. The embedded samples were sectioned into 10 µm‐thick slides, mounted on glass microscope slides, deparaffinized using xylene, and rehydrated following previously established protocols.^[^ ^7^ ^]^ An anti‐myosin polyclonal antibody, produced by immunizing rabbits with purified myosin proteins, was obtained from the Genscript Biotechnology Company (China). This antibody was conjugated with Alexa Fluor 488 NHS Ester (ThermoFisher Scientific, USA). The salivary sheath samples were incubated overnight at 4 °C with a 1:200 dilution of the fluorophore‐conjugated serum. The actin dye phalloidin‐rhodamine (ThermoFisher Scientific, USA) was applied at room temperature with a 1:500 dilution for 30 min, followed by staining with 4′,6‐diamidino‐2‐phenylindole (DAPI) solution (Abcam, USA). Fluorescence images were captured using a Zeiss LSM750 confocal laser‐scanning microscope (Carl Zeiss AG, Germany).

### Observation of Salivary Sheaths Morphology on Parafilm

To assess the effects of dsRNA injection on the salivary sheaths, 2 days post‐injection, nymphs were allowed to feed on an artificial diet for 48 h. The inner Parafilm layers were then removed and placed onto a microscope slide, where the salivary sheaths were counted under a light microscope. For each treatment, 20 SBPHs were used. This experiment included five replicates per treatment and was repeated three times.

Regions of the inner Parafilm layer with salivary sheaths were labeled under a light microscope, and the Parafilm was cut with a scalpel. The salivary sheaths from the Parafilm were attached to a sample holder, coated with gold, and examined with a scanning electron microscope (EVO‐LS10). Five replicates were prepared per treatment, and 20 randomly chosen salivary sheaths were observed.

### Myosin‐rec Infiltration

The *myosin*gene was cloned into a pET‐30a expression vector. *E. coli* strain BL21 (DE3) was transformed with either the pET‐30a‐myosin vector or the pET‐30a empty vector (EV) as a negative control. Protein expression was induced with 1 mM IPTG at 20 °C for 24 h. Cells were harvested by centrifugation, resuspended in phosphate‐buffered saline (PBS; pH 7.5), and lysed with sonication. The lysate was centrifuged at 10 000 × g for 10 min, and the supernatant containing myosin was purified using an AKTA Avant 25 system (GE Healthcare, USA) according to the manufacturer's instructions. The mature recombinant myosin‐rec protein, which includes six N‐terminal His‐tags, has a predicted molecular weight of 18.8 kDa (Figure S15, Supporting Information). Myosin‐rec solutions ranging from 1 nM to 1 µM were infiltrated into the leaves of *N. benthamiana* using a needle‐less syringe. After 12 h, the infiltrated *N. benthamiana* leaves were stained with DAB.

### MP41 Peptide Infiltration

For MP41 infiltration assays, the peptide was synthesized by Genscript Inc (China). The peptide was dissolved in water to obtain a 0.5 mM solution for treating plants. For dicotyledonous plants, the peptide was adjusted to the appropriate concentration and then infiltrated into the leaves using a needle‐less syringe. For monocotyledonous plants, micro‐wounds were created using quartz sand, after which peptide solution was infiltrated into the plants. Peptide solution (0.5 µM) was infiltrated into the leaves of plants of various species, including tomato (*Solanum lycopersicum*), pepper (*Capsicum annum*), maize (*Zea mays*), wheat (*Triticum aestivum*), and cotton (*Gossypium hirsutum*). Photographs of the infiltrated leaves were taken each day at 2 to 7 dpi. For rice stems, a method described previously^[^ ^9^ ^]^ was followed: individual plant stems were punctured 80 times using a #3 insect pin (diameter 450 µm; Beijing Bao Yuan Industrial Technology Co. Ltd., China). Then, 10 µL of 0.5 µM MP41 or water was applied to the newly created wounds.

### VIGS in *N. benthamiana*

VIGS was performed using *A. tumefaciens* strains GV3101 harboring pTRV1 vector and pTRV2:BAK1, pTRV2:SOBIR1, pTRV2:HSP90, pTRV2:NDR1, or pTRV2:EDS1 as described previously.^[^ ^55^ ^]^ The supernatant cells expressing TRV2 constructs were mixed with an *A. tumefaciens* culture expressing TRV1 in a 1:1 ratio in MES buffer (10 mM MgCl_2_, 10 mM MES, 200 µM acetosyringone), to a final OD_600_ of 1.0. The mixed cell suspensions were then infiltrated into three primary leaves of four‐leaf‐stage *N. benthamiana* plants using a 1 mL needleless syringe. As a visual indicator of the progression of virus infection, a previously described pTRV2 with a fragment of the phytoene desaturase gene that causes an albino phenotype 10–14 days after infiltration was used as a positive infiltration control. pTRV2:GFP was used as a negative control. After 3 weeks, the third newly emerged leaves were harvested for RNA extraction and RT‐PCR analysis to validate the silencing efficiency. *Agrobacterium* strains harboring elicitors were then infiltrated into the third newly emerged leaves of VIGS‐silenced plants. Photos were taken at 4 dpi.

### Cell Death and ROS Bursts Inhibition Assays by Using LaCl_3_ or Transient Expression of CaM

The effects of the calcium channel inhibitor LaCl_3_ on cell death and ROS bursts were determined as previously described.^[^ ^6^ ^]^ Briefly, 100 nM myosin‐rec was mixed with 1 mM LaCl_3_ and infiltrated into *N. benthamiana* leaves. At 12 hpi, the infiltrated leaves were stained with DAB. The role of CaM in myosin‐rec‐triggered cell death and ROS bursts was evaluated as previously described.^[^ ^25^ ^]^ Briefly, empty pBINPLUS‐GFP or pBINPLUS‐GFP harboring CaM were transformed by electroporation into *A. tumefaciens* GV3101. The *A. tumefaciens* was resuspended in an infiltration buffer (10 mM MgCl_2_, 500 mM MES, and 100 mM acetosyringone). At a final OD_600_ of 0.4, the *A. tumefaciens* was infiltrated into *N. benthamiana* leaves. CaM‐GFP was expressed for 24 h, after which 100 nM myosin‐rec was infiltrated into the same region. At 12 hpi, the infiltrated leaves were stained by DAB.

### ROS Assays

Leaf disks collected from 4‐week‐old *N. benthamiana* or *Nipponbare* leaves were harvested and then soaked in water to keep in the dark at room temperature. The leaf disks were stained in a test buffer containing luminol (Sigma, USA), L012 (Fujifilm WAKO, Japan), horseradish peroxidase (Sigma, USA), and 0.5 µM flg22 (GenScript, China) or 0.5 µM myosin‐rec/EV or 0.5 µM MP41/water. Luminescence detection was conducted to measure ROS production (BioTek, China).

### MAPK Phosphorylation

Total proteins were extracted from 4‐week‐old *N. benthamiana* leaves after treatment with 0.5 µM flg22, myosin‐rec/EV or MP41/water. Isolated proteins were separated on a 12% (v/v) SDS–PAGE gel for immunoblotting. Phosphorylation of MAPK proteins including MPK3, MPK4, and MPK6 was determined using anti‐phospho‐p44/42

MAPK antibody (1:5000, Cell Signaling Technology, USA) and HRP‐conjugated anti‐rabbit IgG (1:10000, Sigma, USA) as a secondary antibody for immunoblot detection.

### Western Blotting

The leaves of *N. benthamiana* were ground into powder. Subsequently, 2 mL of NP40 buffer (Beyotime Institute of Biotechnology, China) was added, followed by vortexing at 4 °C for 20 min. Each sample was then centrifuged at 15200 × g and 4 °C for 5 min. After centrifugation, the supernatant was collected and concentrated to 200 mL using a YM‐3 Microcon centrifugal filter device (EMD Millipore, USA). The samples were analyzed using SDS‐PAGE on a 12% (w/v) gradient gel (Bio‐Rad Laboratories, USA) and transferred to a PVDF membrane. Western blotting was performed using anti‐GFP (1:2000 dilution) followed by goat anti‐mouse (1: 8000 dilution) antibodies.

In the RSV assay, total proteins of the mock‐ or virus‐inoculated rice seedlings were extracted from the plants, heated, and resolved with SDS‐PAGE. The RSV NS3 protein was assessed using the polyclonal antiserum generously provided by Prof. Zhen He (Yangzhou University), followed by a secondary AP‐linked antibody. Bands were visualized using NBT/BCIP (Sangon Biotech, China) or Clarity Western ECL Substrate (Bio Rad, USA).

### Generation of Myosin‐Expressing Rice Lines

The *myosin* was ligated into the vector pCAMBIA1301 (Cambia, USA) under the control of the CaMV 35S promoter. The T‐DNA was introduced into the genome of *Nipponbare* rice using *A. tumefaciens* mediated transformation. Homozygous T_2_ plants were selected using hygromycin resistance and GUS staining.

### Analysis of SA, JA, JA‐Ile, and H_2_O_2_

Rice sample preparation for phytohormone and H_2_O_2_ analyses followed established protocols.^[^ ^30^ ^]^ For myosin‐expressing and WT rice, the rice stems were placed into a glass cylinder containing 20 newly emerged SBPH female adults. After 8 or 24 h, the SBPHs were removed and the stems were collected. For rice treated with exogenous MP41 or water, micro‐wounds were created using quartz sand, and then 0.5 µM MP41 or water was infiltrated into the wounded rice stem. The outer three leaf sheaths in the treated rice stems were collected after 4 or 8 h. Samples were ground in liquid nitrogen, extracted with ethyl acetate containing labeled internal standards (^2^D_4_‐SA, ^2^D_6_‐JA, and ^2^D_6_‐JA‐Ile), and analyzed for SA, JA, and JA‐Ile content using high‐performance liquid chromatography‐tandem mass spectrometry. H_2_O_2_ concentrations were quantified using the Amplex Red hydrogen peroxide/peroxidase assay kit (ThermoFisher Scientific, USA).^[^ ^56^ ^]^ Each experiment was repeated 5 to 6 times, with 3 plant samples per replicate.

### Olfactometer Bioassays

The behavioral responses of newly emerged *A. nilaparvatae* females (0‐24 h post‐ecdysis) to rice volatiles were assessed using a Y‐tube olfactometer following previously described methods.^[^ ^45^ ^]^ The attraction of parasitoid females to the following pairs of odor sources was recorded. Rice plants treated with MP41 for 12 h versus plants treated with water; and myosin ‐expressing lines versus WT plants. Seven plants were used per treatment, and the odor sources were replaced with new plants after testing 20 wasps. In total, seven sets of plants and 140 female parasitoids were used.

### Collection and Isolation of Volatiles

Volatiles emitted from individual plants were collected for 8 h, isolated, and identified as described previously, with some modifications.^[^ ^45^ ^]^ The headspace collection of rice volatiles was carried out by placing a rice plant as a single odor source into a 3142 mL gas collection bottle. Air was passed through activated carbon, molecular sieves, and red silica gel filters before entering through the upper connection of the glass collection bottle. The bottom connection was connected to a tube containing 30 mg of Super Q adsorbent (ANPEL Laboratory Technologies, China), and the flow rate was controlled by a flow meter at 500 mL min^−1^. Prior to gas collection, the flow rate was maintained for 30 min to purge any impurities in the bottle. Volatiles were collected in an artificial climate chamber at 26 ± 2 °C and 75 ± 10% humidity for 8 h (from 9:00 to 17:00). After collection, 600 µL of n‐hexane was used to elute the volatiles into a sample vial. The collected samples were stored in a 4 °C refrigerator for later analysis. Compounds were quantified as a percentage of peak area relative to the internal standard (diethyl sebacate) per 8 h of trapping session. Collections were repeated six times per treatment.

### Field Experiments

Seeds of WT plants and those expressing myosin were germinated and grown under the conditions described previously. At 30 d of cultivation in a glasshouse following germination, the plants were transferred to a field plot at the experimental field of Jiangsu Academy of Agricultural Sciences in Nanjing, China. The rice plants were covered with mesh netting, which measured ≈10 m in length, 3 m in width, and 4 m in height, designed to prevent the insects from escaping the experimental area. The planthoppers, including 500 *N. lugens* nymphs, 500 *L. striatellus* nymphs, and 500 *Sogatella furcifera* nymphs were then released into the field. The total number of planthoppers per plant, including both nymphs and adults, was counted at 40, 54. and 68 d after planting.

### *Suppressalis* Weight Test

After weighing, two second‐instar *C. suppressalis* larvae were placed in each glass tube with holes and attached to each plant basal stem. After 7 day of feeding, each insect was reweighed to assess weight gain. Weighing was performed using an electronic balance (Mettler Toledo, Switzerland). This experiment included twenty insects per treatment and was repeated three times.

### Rice Blast Fungal Inoculation

For *M. oryzae* inoculation, two‐week‐old rice seedlings were sprayed with a suspension of 5 × 10^4^/mL spores of *M. oryzae* strain Guy11(ATCC201236) in a 0.2% (w/v) gelatin solution and kept wet at 28 °C. Disease symptoms were scored 7 day after infection by measuring the lesion areas and disease indexes. Disease indexes were evaluated based on 10 classes from 0 (no disease lesions) to 9 (more than 75% disease lesions) as previously described elsewhere.^[^ ^57^ ^]^ The lesion area (%) was quantified digitally by photographing the area and then calculating the number of pixels under the lesion and healthy areas of diseased leaf blades using the Histogram command in Adobe Photoshop.^[^ ^58^ ^]^ The experiment was repeated three times.

### Artificial Inoculation of RSV

For RSV inoculation, SBPHs carrying RSV were transferred to rice seedlings cultured in a 1 L glass beaker. After 3 day of feeding, the seedlings were divided and transplanted into a 20 L plastic bucket. For the mock treatment, non‐virulent SBPHs were transferred to a glass beaker and were cultured with the rice seedlings for 3 day, after which the seedlings were transplanted in the same manner as the RSV‐inoculated seedlings. The young rice plants were then covered with pest control nets and were cultured in a growth chamber. After 45 days, total protein was extracted from the rice seedlings and analyzed. Western blotting was performed to assess the presence of RSV, and the presence or absence of RSV infection was confirmed using RT‐PCR at 30 dpi. The severity of the symptoms on new leaves was graded, and representative photographs were taken (Figure S12, Supporting Information). Specific primers for viral testing are listed in Table S3 (Supporting Information). The experiment was repeated three times.

### *Xoo* Virulence Assay

The *Xoo* train PXO99A and its derivatives were grown at 28 °C in nutrient broth (NB) or on NB agar (NA) plates. A single colony was inoculated in a liquid lysogeny broth medium and incubated at 37 °C with shaking at 220 rpm for 10 h. The bacterial suspension was then diluted to ≈10^9^ colony‐forming units (CFU) mL^−1^ using sterile distilled water. Scissors were immersed in the bacterial suspension and used to cut 2 cm of rice plant leaves. After inoculation, the rice plants were maintained in a controlled climate box (30 ± 1 °C, 90 ± 5% RH, and 20 000 lx light intensity) for 12 h. Subsequently, the plants were transferred to a greenhouse and grown under the same conditions. Disease symptoms were recorded at 7 and 14 dpi, and lesion lengths were measured. The experiment was repeated three times.

### Insect Bioassays on *N. benthamiana* and *G. hirsutum*

In the choice test, eight second‐instar *S. frugiperda* larvae, fifteen newly emerged *B. tabaci* female adults, eight second‐instar *H. armigera* larvae, or fifteen *A. gossypii* adults were introduced to the center of each host leaf pair treated either with 0.5 µM MP41 or water. The number of insects on each leaf was recorded at 1, 2, 4, 8, 12, 24, and 48 h. This experiment included ten replicates per treatment and was repeated three times.

In the no‐choice test, *B. tabaci* fecundity assays were conducted following Atamian et al.^[^ ^59^ ^]^ with slight modifications. *N. benthamiana* leaves infiltrated with MP41 or water were placed in individual wells of a 24‐well plate. Four first‐instar *B. tabaci* nymphs were placed on each leaf disc. A pair of *B. tabaci* adults was placed on each leaf. After 2 and 4 days, the insects were transferred to new MP41‐treated or water‐treated leaves. The number of eggs was recorded on days 2, 4, and 6. This experiment consisted of twelve replicates per treatment and was repeated three times. Similarly, cotton leaves infiltrated with MP41 or water were placed in culture dishes. Four *A. gossypii* adults were placed on each leaf, and after 3 days, new offspring were counted under a microscope. This experiment included fifteen replicates per treatment and was repeated three times.

### Statistical Analysis

Differences among treatments were analyzed with one‐way ANOVA followed by Duncan's multiple range test to compare treatments (or a student's *t*‐test when only two treatments were compared). Data analyses were performed using SPSS STATISTICS 19.

## Conflict of Interest

The authors declare no conflict of interest.

## Author Contributions

R.J., L.‐X.Q., and J.‐C.F. planned and designed the research. L.‐X.Q., J.L., S.L., J.L., H.W., L.Y., X.‐Y.T., and Z.‐C.Z. performed experiments and analyzed data. G.‐H.L. and M.‐F.J. provided valuable suggestions for the research. L.‐X.Q. and R.J. drafted the manuscript, L.‐X.Q., A.‐A.H., J.‐C.F., and R.J., revised the manuscript.

## Supporting information



Supporting Information

## Data Availability

Sequence data are available in NCBI under the following accession numbers: myosin (RZF45242.1), actin related protein 1 (RZF32723.1), NbBAK1b (G14GQ‐62524, Ni‐ben101Scf02513g11004.1), OsBAK1 (NP_001409376.1), and OsMYC2 (XP_025876009.1). The data that support the findings of this study are available from the corresponding author upon reasonable request.
