# Supplementary material for: An Insect Salivary Sheath Protein Triggers Plant Resistance to Insects and Pathogens as a Conserved HAMP
Source: Adv Sci (Weinh). 2025 Apr 1;12(21):2415474. doi: 10.1002/advs.202415474 (PMC12140304; doi:10.1002/advs.202415474)
Supplement: Supplementary file 1 — Supporting Information [file ADVS-12-2415474-s001.docx]

**Supporting Information**


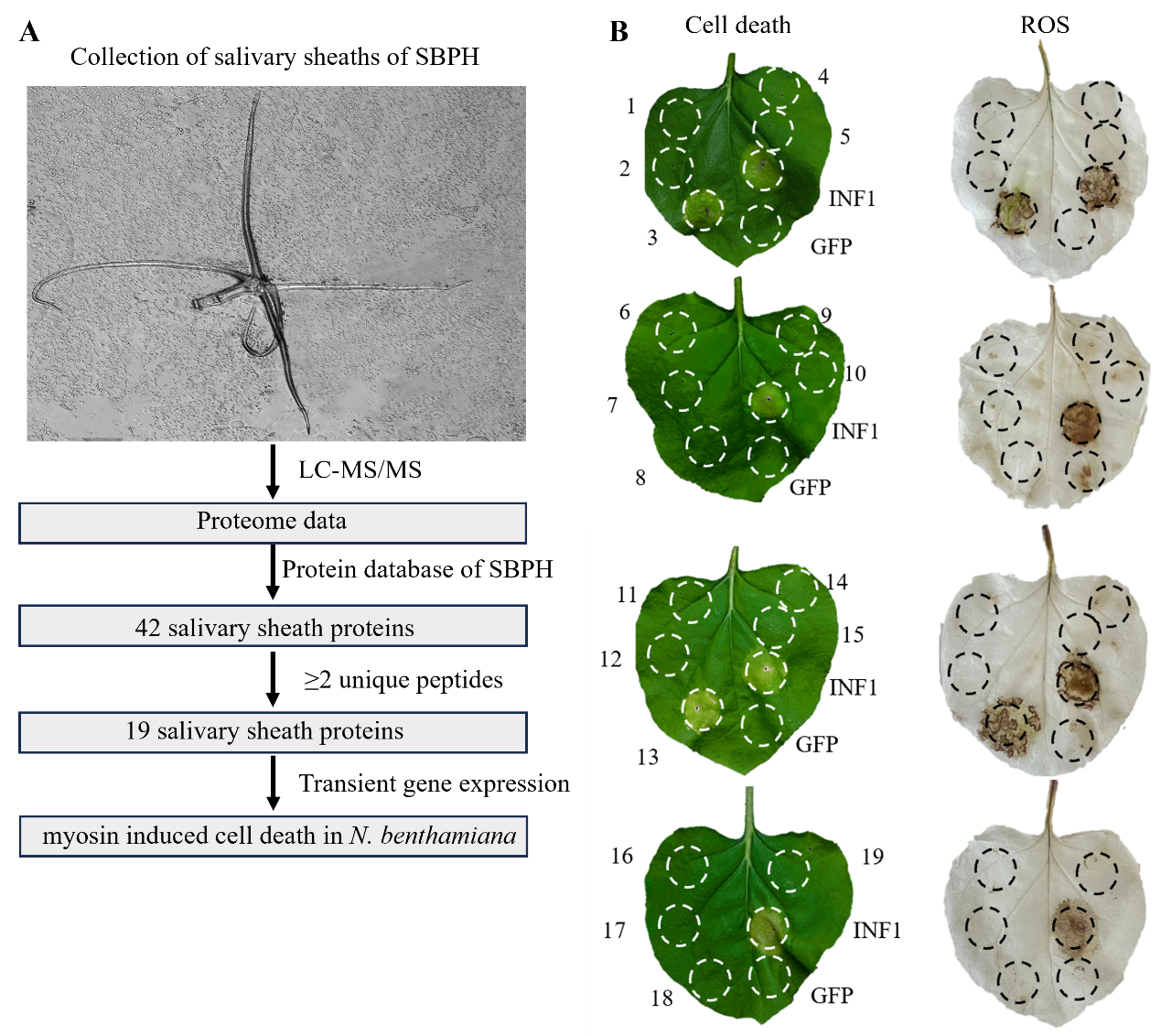


**Fig. S1 myosin and actin related protein 1 from SBPH salivary sheaths significantly induced cell death and ROS production**. (A) Pipeline for the identification of candidate elicitors. (B) *N. benthamiana* leaves were infiltrated with *Agrobacterium* carrying either GFP or indicated genes fused to GFP. The left and right panel depict detection of cell death and ROS production in *N. benthamiana*. The experiment was repeated with 10 leaves. 1, myosin heavy chain. 2, paramyosin long form-like. 3, actin related protein 1. 4, mitochondrial ATP synthase subunit alpha. 5, arginine kinase. 6, tropomyosin-1. 7, histone H4-like. 8, carboxylase/oxygenase large subunit. 9, tropomyosin 1. 10, elongation factor 1-alpha. 11, ATP synthase subunit beta. 12, calcium-transporting ATPase. 13, myosin light chain 1-like. 14, myosin regulatory light chain 2. 15, histone H2B. 16, alpha-actinin. 17, actin. 18, ADP/ATP translocase. 19, tubulin beta-1 chain. INF1/GFP served as the positive/negative controls. Photos were taken at 2 dpi.


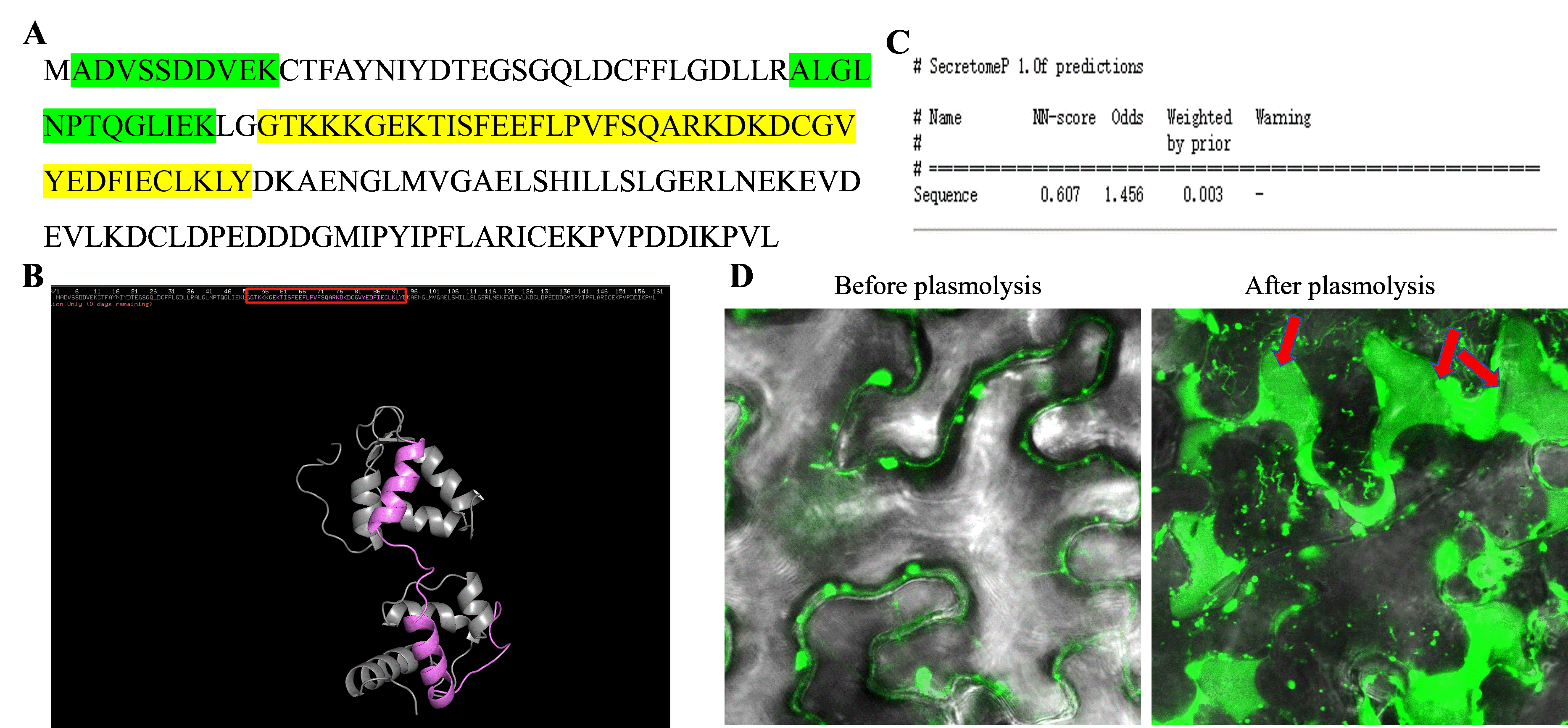


**Fig. S2 Characteristics of myosin proteins.** (A) Amino acid sequence of myosin, green shading indicates two unique peptides detected by LC-MS/MS, while yellow shading marks the minimal immunogenic epitope responsible for eliciting immune responses, a 41-amino-acid peptide (MP41). (B) Predicted protein structure by AlphaFold3 with PyMOL visualization. MP41 is highlighted in pink. (C) The prediction of myosin as an unconventionally secreted protein using SecretomeP software. The NN-score of myosin is higher than the recommended threshold (0.5-0.6). (D) The subcellular localization of myosin in *N. benthamiana* cells before and after plasmolysis. The localization of myosin-GFP in the plant apoplast was indicated by arrow. Mannitol solution (0.8 M) was carefully applied to the leaf sections. After incubation for 5 min, the leaf sections were examined under a confocal fluorescence microscope to observe the plasmolysis in detail.


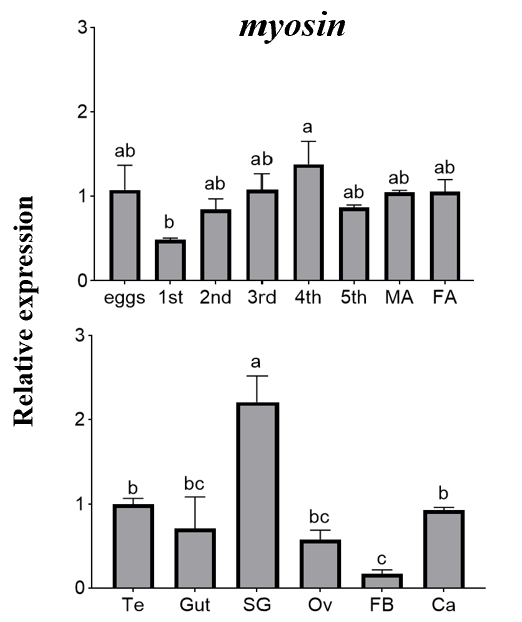


**Fig. S3 Temporal and spatial expression profile of *myosin* in SBPH detected by RT-qPCR.** Upper panel, mean transcript levels (+ SE, *n* = 3) of *myosin* in whole insects at different developmental stages. 1st to 5th instar nymphs; MA, male adult; FA, female adult. Lower panel, mean transcript levels (+ SE, *n* = 3) of *myosin* in different tissues. Te, testes; SG, salivary gland; Ov, ovary; FB, fat body; Ca, carcass. Different letters indicate significant differences among treatments (P < 0.05, one-way ANOVA followed by Duncan’s multiple range test).


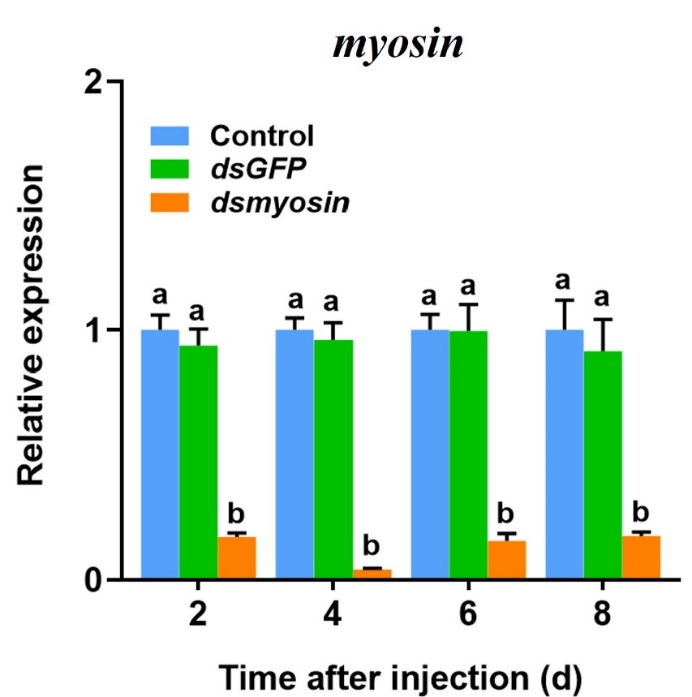


**Fig. S4 The efficiency of RNAi via dsRNA injection** **detected by RT-qPCR**. Mean transcript levels (+ SE, *n* = 3) of *myosin* in the third-instar nymph stage of SBPH injected with *dsmyosin*, *dsGFP* or noninjected. Different letters indicate significant differences among treatments (P < 0.05, one-way ANOVA followed by Duncan’s multiple range test).


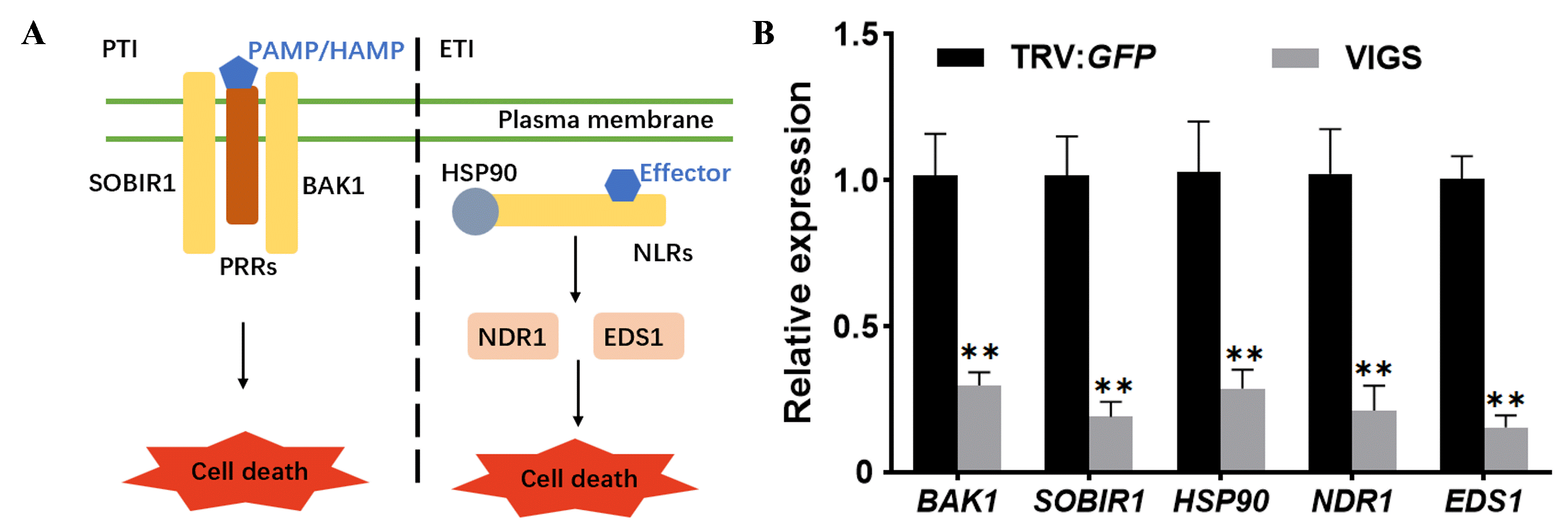


**Fig. S5 The efficiency of VIGS in** ***N. benthamiana*** **detected by RT-qPCR.** (A) A diagram illustrating PTI- and ETI-mediated immunity. PTI is triggered when PAMP/HAMPs are recognized by PRRs interacting with BAK1 and/or SOBIR1 on the plant cell membrane. For ETI, effectors are recognized by intracellular NLRs, which require HSP90. EDS1 and NDR1 are essential for NLR-mediated immune responses. (B) Silencing efficiency (+ SE, *n* = 3) of *NbBAK1*, *NbHSP90*, *NbSOBIR1*, *NbEDS1*, *and NbNDR1* in the VIGS-treated *N. benthamiana* plants. Asterisks indicate significant difference between treatments (**, *P* < 0.01; Student’s *t*-tests).


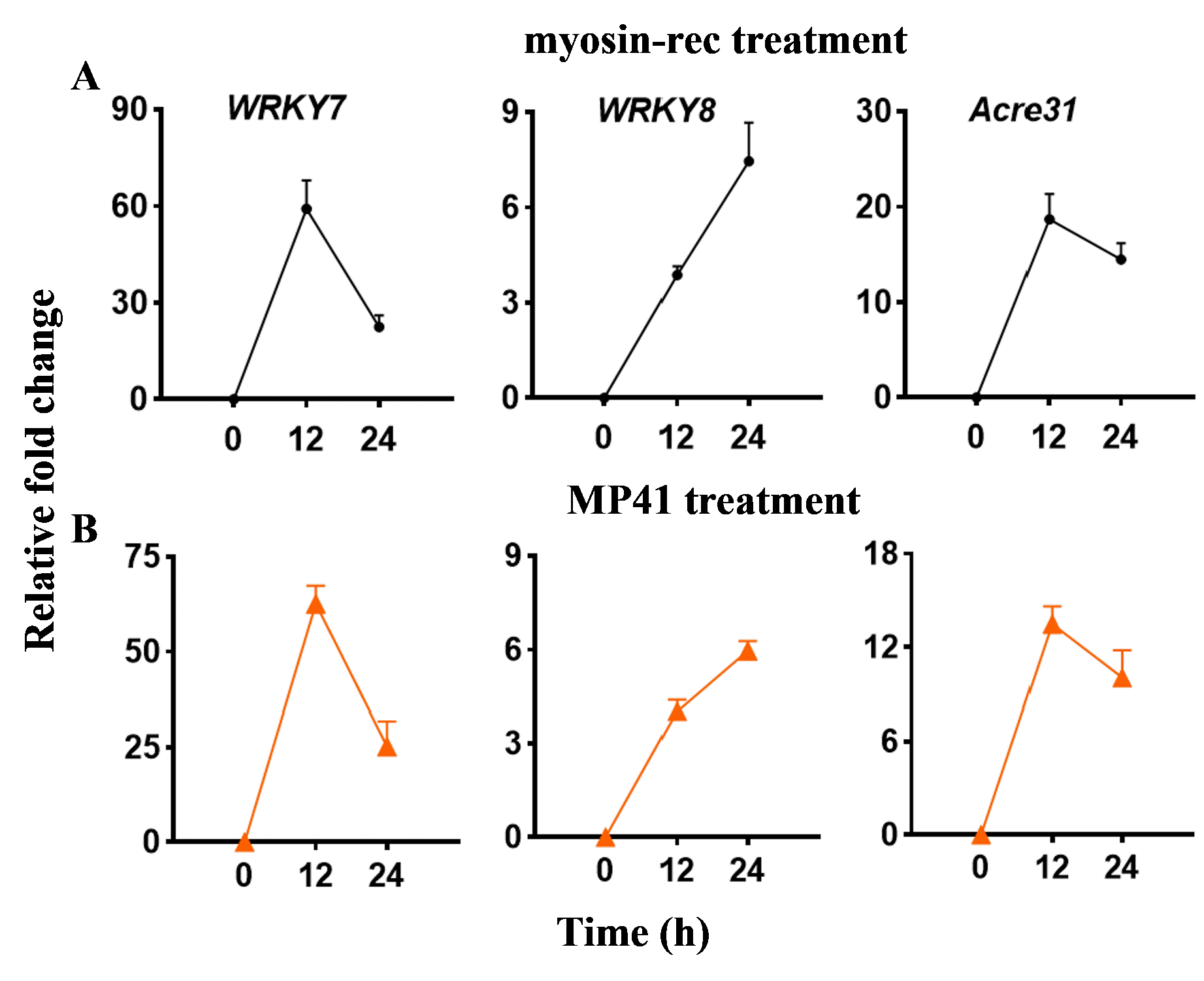


**Fig. S6** **Myosin-rec /MP41 triggered the upregulation of PTI marker genes in *N. benthamiana*.** (A-B) Myosin-rec (A) and MP41 (B) treatment induced the upregulation of PTI marker genes. Mean fold change (+ SE, *n* = 3) were quantified by RT‐qPCR.


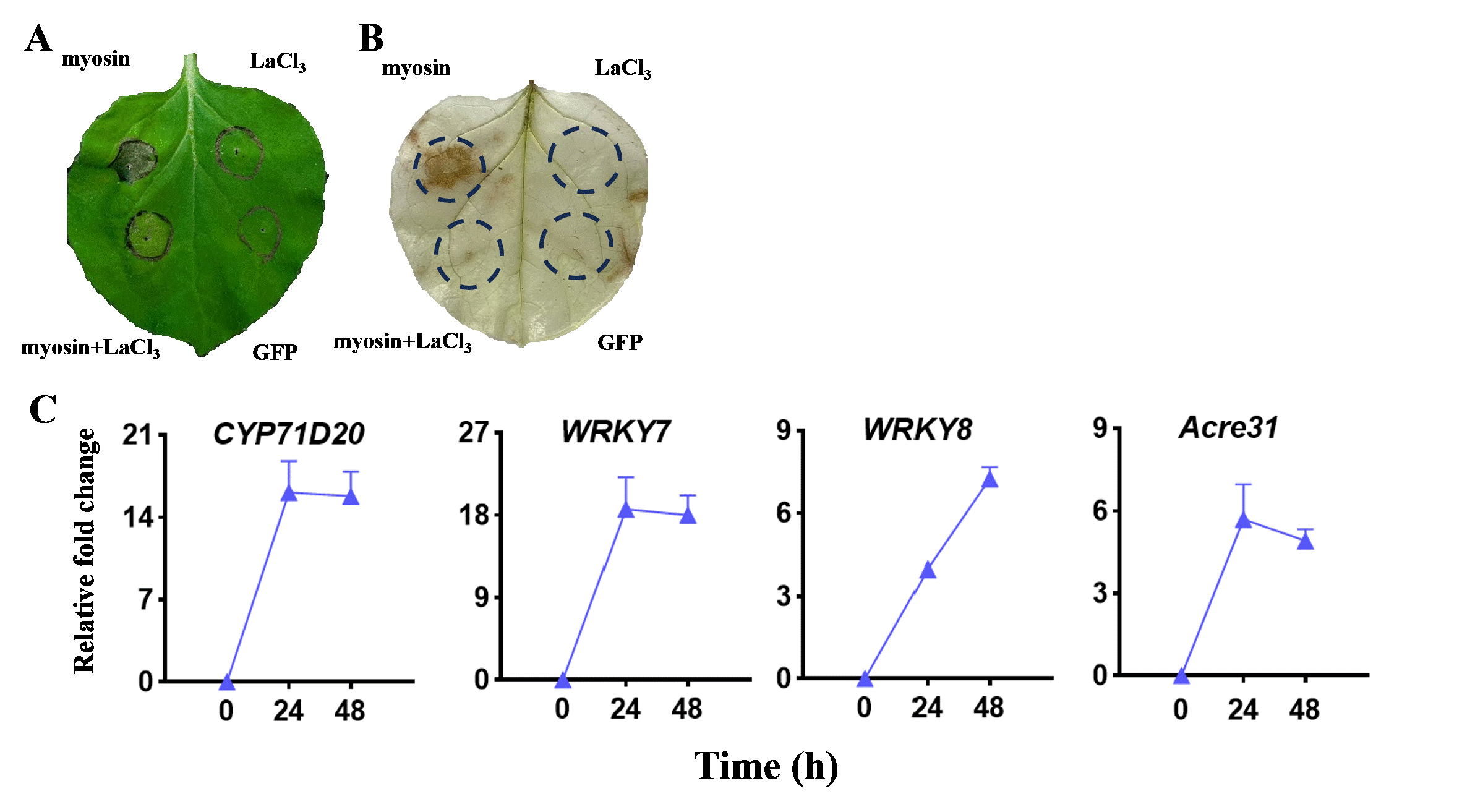


**Fig. S7 Myosin induces PTI responses in *N. benthamiana*.** (A-B) The inhibition of the calcium channel inhibitor LaCl_3_ on plant cell death (A) and ROS production (B) induced by myosin. The inhibition effect was observed in all 20 experimental leaves of *N. benthamiana*. Photos were taken at 2 dpi. (C) Myosin triggered the upregulation of PTI marker genes. Mean fold change (+ SE, *n* = 3) were quantified by RT‐qPCR.


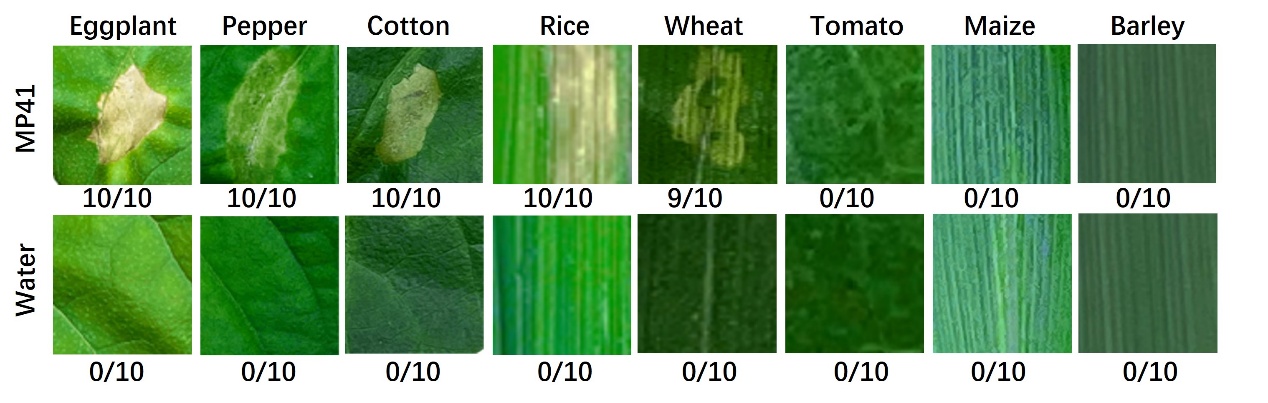


**Fig. S8 Cell death in multiple plant species triggered by exposure of 0.5 μM MP41 or water control.** The experiment was repeated with 10 leaves. Photos were taken at 7 dpi.


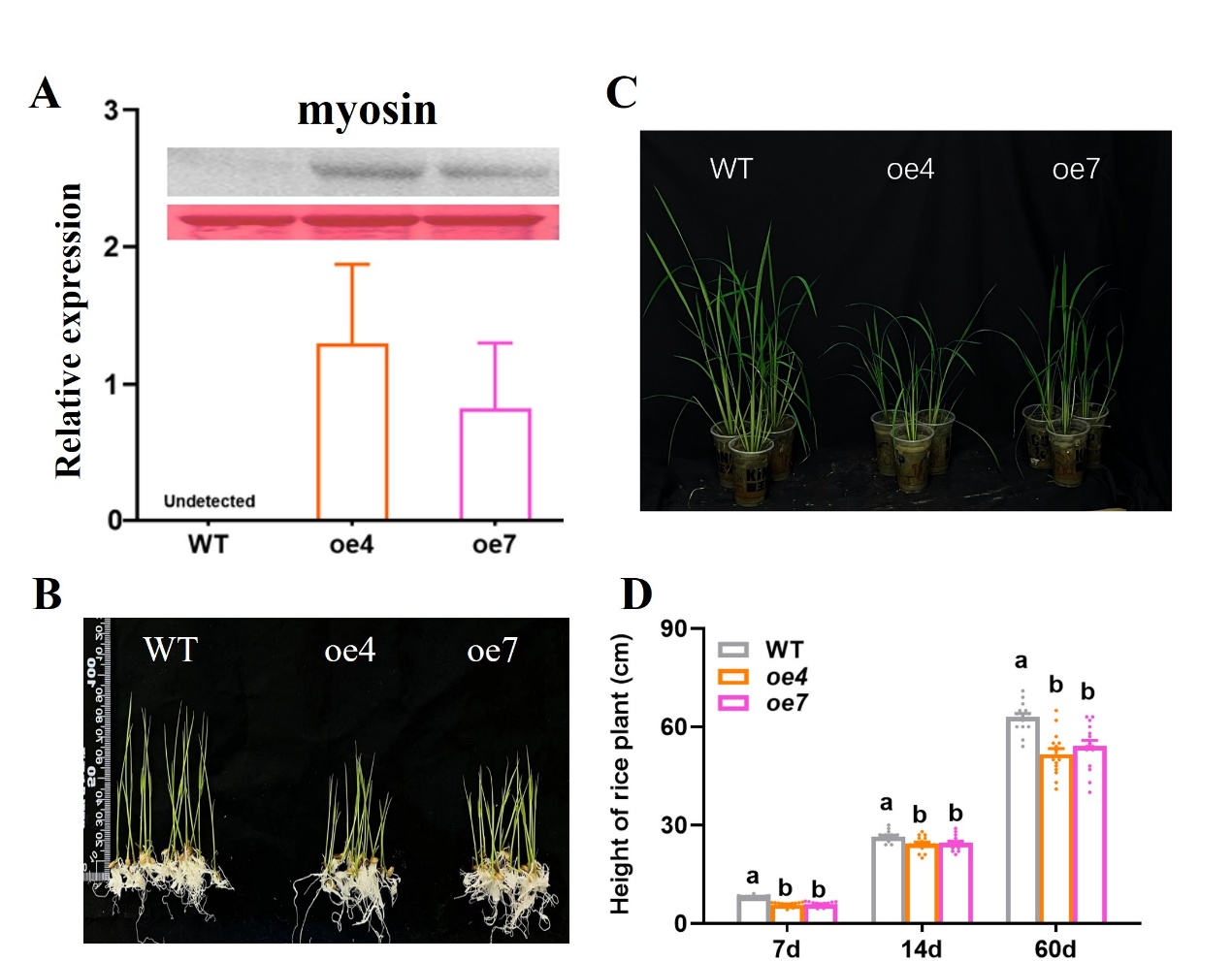


**Fig. S9 Expression of myosin and growth status of *myosin*-expressing rice lines**. (A) Mean expression levels (+ SE, *n* = 3) of the *myosin* gene in WT, oe4, and oe7 rice. Insert: Protein levels of the myosin in WT, oe4, and oe7 rice. Protein loading was indicated by Ponceau S staining for Rubisco protein. (B) Typical images of the growth status of oe4, oe7, and WT rice seeds 7 days after germination. Each observation group consists of 3 rice plants. (C) Typical images of the growth status of oe4, oe7, and WT rice seeds 50 days after germination. Each observation group consists of 15 rice plants. (D) Mean height (+ SE, *n* = 24) of oe4, oe7, and WT rice plants at 7, 14, and 60 days after seed germination. Different letters indicate significant differences among treatments (P < 0.05, one-way ANOVA followed by Duncan’s multiple range test).


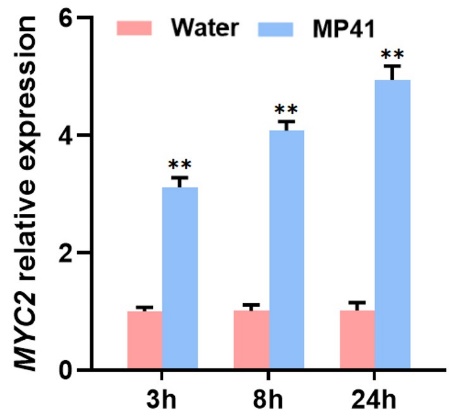


**Fig. S10 MP41 upregulated *MYC2* expression in rice**. Mean expression levels (+ SE, *n* = 3) of *MYC2* induced by 0.5 μM MP41 or water in rice at 3, 8, 24 h. Asterisks indicate significant difference between treatments (**, *P* < 0.01; Student’s *t*-tests).


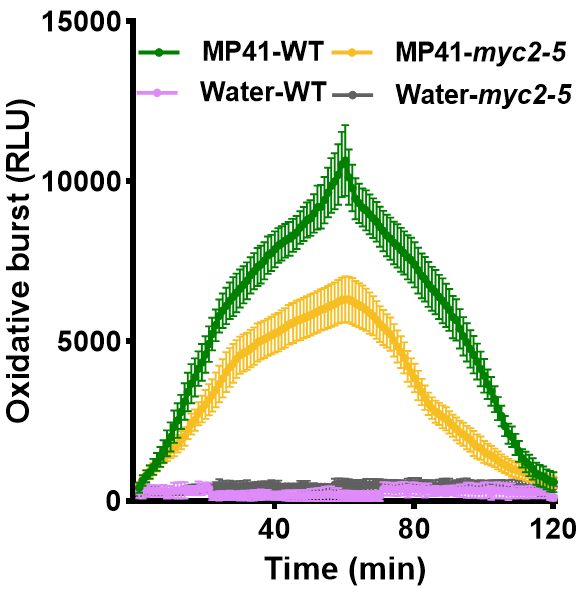


**Fig. S11 *myc* knockdown slightly reduces MP41-induced ROS bursts in rice**. ROS burst in WT and *myc2-5* rice plant leaves challenged with 0.5 μM MP41 or water. ROS production was measured with a luminol-based assay. Mean RLU [Relative Luminescence Unit (± SE, n = 6)] are shown.


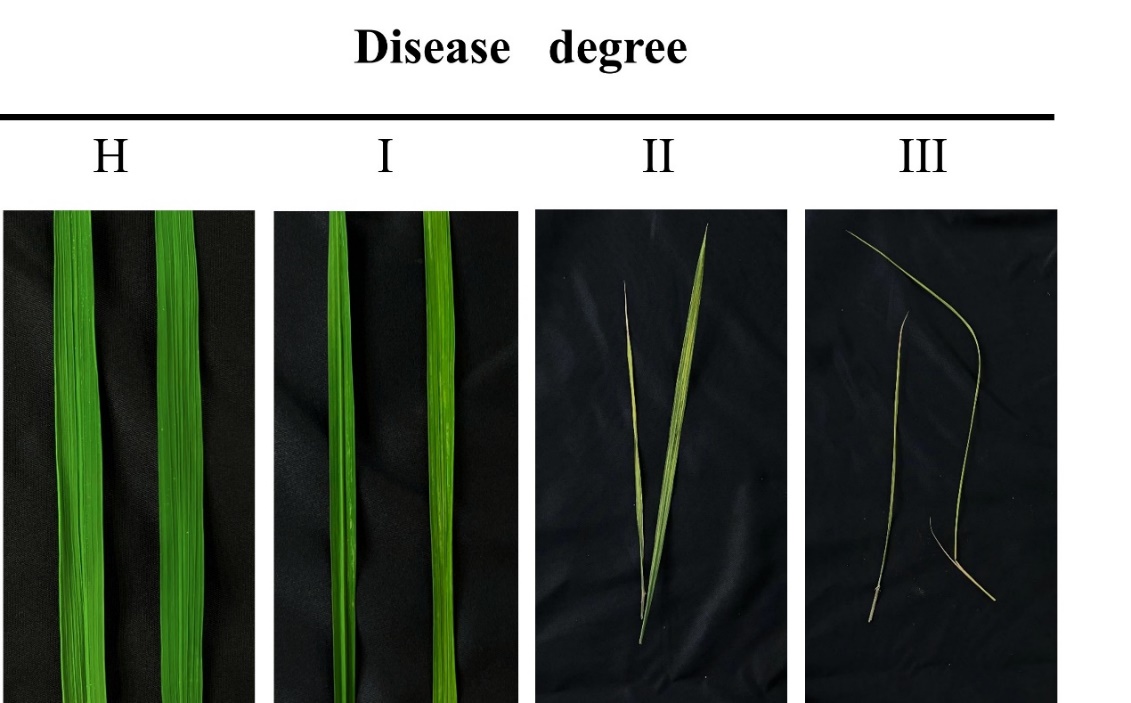


**Fig. S12 Typical disease symptoms (grade I to grade III) of RSV-infected rice plants.** H: heathy plants; I: milder virus symptoms with discontinuous yellow stripes and necrotic streaks; II: typical yellow stripes and necrotic stripes; III: severe curling or death of the young leaves.


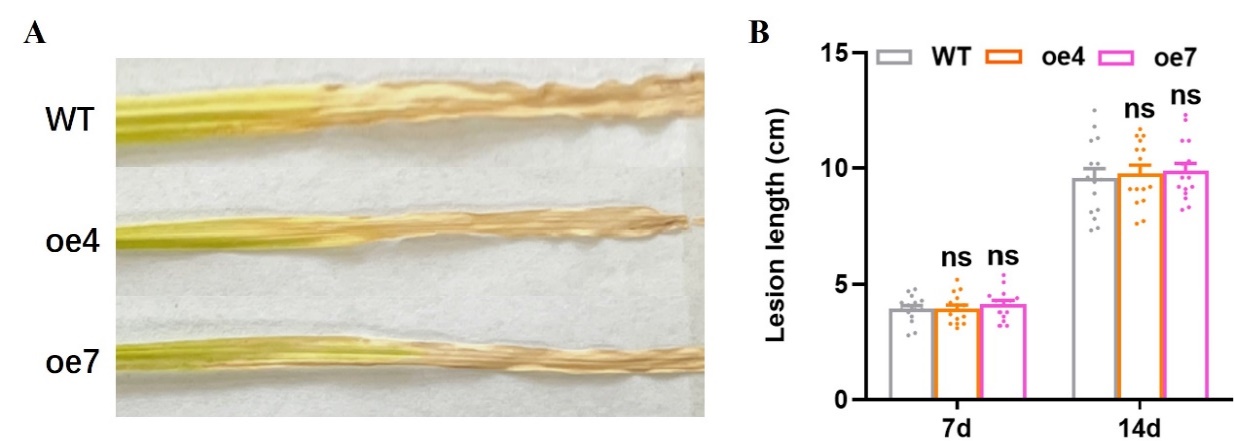


**Fig. S13 Typical symptoms and lesion length of rice bacterial blight.** (A) Symptoms of bacterial blight on rice leaves photographed at 14 days. The experiment was repeated with 30 rice leaves. (B) Mean blight lesion length (+ SE, *n* = 15) at 7 and 14 dpi. Ns, not significant (Student’s *t*-tests).


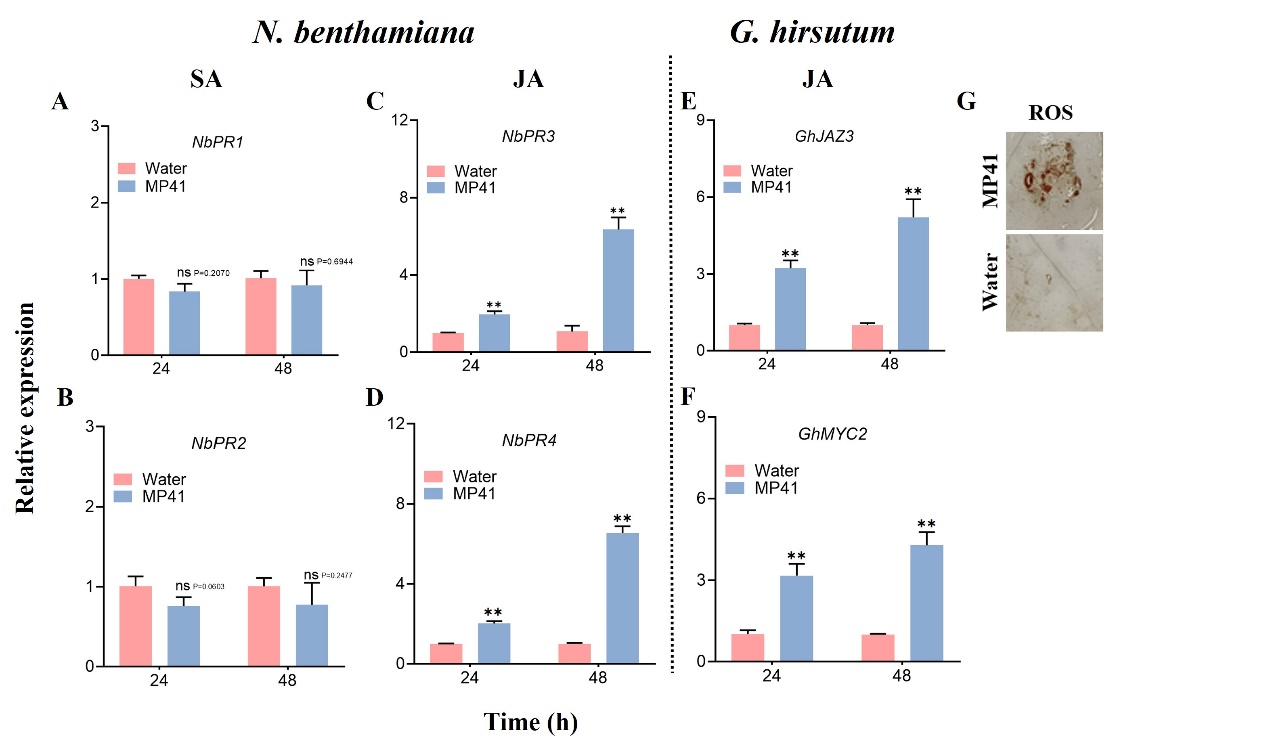


**Fig. S14 MP41 induced JA marker genes in *N. benthamiana* and** ***G. hirsutum***. Left panel, mean expression levels (+ SE, *n* = 3) of *NbPR1* (SA-related gene; A) and *NbPR2* (SA-related gene; B), *NbP*R3 (JA-related gene; C), and *NbPR4* (JA-related gene; D) in *N. benthamiana* leaves treated by 0.5 μM MP41 or water at 24 and 48 h. Right panel, mean levels (+ SE, *n* = 3) of *GhJAZ3* (JA-related gene; E) and *GhMYC2* (JA-related gene; F) and ROS production (G) in *G. hirsutum* leaves treated by 0.5 μM MP41 or water at 24 and 48 h. The experiment was repeated with 10 leaves in (G). Asterisks indicate significant difference between treatments (**, *P* < 0.01; Student’s *t*-tests).


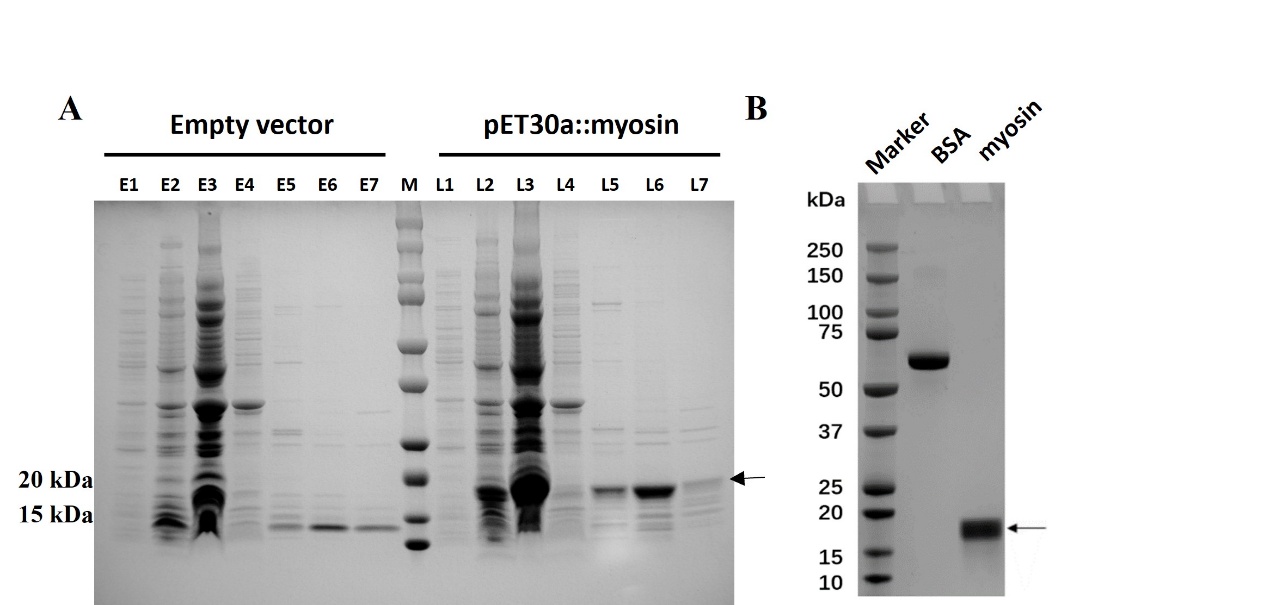


**Fig. S15 SDS-PAGE analysis of purified recombinant myosin.** (A) SDS-PAGE analysis of proteins collected from the following purification steps. E1/L1: Uninduced whole cells, E2/L2: induced whole cells, E3/L3: Induced cell lysis supernatant, E4/L4: Induced cell lysis precipitation, E5-E7: Purified EV protein, L5-L7: Purified myosin protein. (B) Western blotting of the purified recombinant myosin. BSA used as a control. Recombinant His-myosin is indicated by arrow. M: marker.


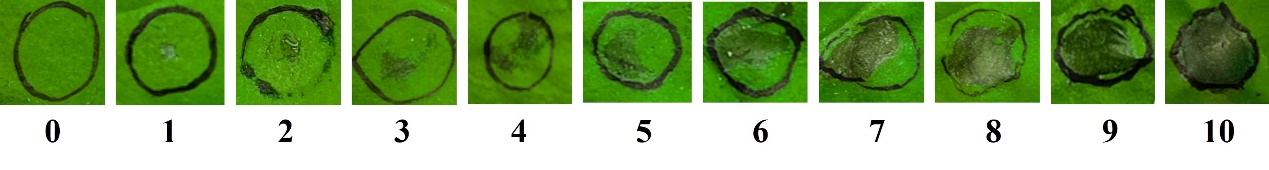


**Fig. S16 Illustration of the cell death index.** Images showing cell death phenotypes at scales ranging from 0 (no cell death phenotype) to 10 (fully confluent necrosis).

**Table S1. Identification of SBPH Salivary Sheath Proteins**

| Protein | GeneBank ID | Unique peptides |
| --- | --- | --- |
| Myosin heavy chain | APA34054.1 | 34 |
| Paramyosin long form-like | RZF40854.1 | 12 |
| Actin related protein 1 | RZF32723.1 | 10 |
| Mitochondrial ATP synthase subunit Alpha | AIL26069.1 | 5 |
| Arginine kinase | RZF41846.1 | 4 |
| Tropomyosin-1 | RZF37477.1 | 3 |
| Histone H4-like | XP_015372596.1 | 3 |
| Carboxylase/oxygenase large subunit | ASD35392.1 | 3 |
| Tropomyosin 1 | RZF46537.1 | 3 |
| Elongation factor 1-alpha | RZF35682.1 | 3 |
| ATP synthase subunit beta | RZF41650.1 | 3 |
| Calcium-transporting ATPase | RZF36560.1 | 2 |
| Myosin light chain 1-like | RZF45242.1 | 2 |
| Myosin regulatory light chain 2 | RZF41800.1 | 2 |
| Histone H2B | AAB48092.1 | 2 |
| Alpha-actinin | RZF39134.1 | 2 |
| Actin | GFT55090.1 | 2 |
| ADP/ATP translocase | RZF32594.1 | 2 |
| Tubulin beta-1 chain | XP_022207921.1 | 2 |
| Tropomyosin-1 | XP_022187623.1 | 1 |
| Uncharacterized protein LOC111032217 | XP_022168166.1 | 1 |
| Chlorophyll A/B binding protein 1 | NP_174286.1 | 1 |
| Hypothetical protein LSTR_LSTR003418 | RZF39757.1 | 1 |
| Hypothetical protein LSTR_LSTR004975 | RZF47266.1 | 1 |
| Dynein beta chain | RZF44437.1 | 1 |
| Splicing factor 1 | KAF7742323.1 | 1 |
| Photosystem II CP43 reaction center protein | KAH9752498.1 | 1 |
| Putative secretory protein | QMU23604.1 | 1 |
| Histone H4 | CAF9928778.1 | 1 |
| Hypothetical protein DSO57_015870 | KAF7751974.1 | 1 |
| Cuticle protein 7-like | XP_022186634.1 | 1 |
| ATP synthase subunit alpha | KXN69528.1 | 1 |
| Beta subunit of the F1 sector of mitochondrial F1F0 ATP synthase | RCI05405.1 | 1 |
| Actin | AMZ01553.1 | 1 |
| LSTR_LSTR007434 | RZF40234.1 | 1 |
| Heat shock protein 70-5 | AQP31338.1 | 1 |
| Tubulin alpha-1 chain | RZF47578.1 | 1 |
| Tropomyosin-1 | RZF41119.1 | 1 |
| Vacuolar protein sorting-associated protein 8 homolog | XP_022195458.1 | 1 |
| ATP synthase lipid-binding protein | RZF46192.1 | 1 |
| BTB/POZ domain-containing protein 1 | RZF37421.1 | 1 |
| LSTR_LSTR011913 | RZF33379.1 | 1 |

**Table S2. Primers used for genes clone**

| **Primers** | **Sequences (5’-3’)** |
| --- | --- |
| myosin-F | ATGGCAGATGTCAGCAGCGATG |
| myosin-R | TTACAATACTGGCTTGATGTC |
| Nlmyosin-F | ATGGCTGATGTGAGCAGTGATG |
| Nlmyosin-R | TTACAATACTGGCTTGATGTC |
| Asmyosin-F | ATGGCCCACGATCTGAAGGATG |
| Asmyosin-R | TTACGGTGCGTGGTCACACA |
| Almyosin-F | ATGAGCCAGGTCCGTAGGCCAG |
| Almyosin-R | TCAAGGTATGATGGAGTCAG |
| Btmyosin-F | ATGGCCGATCTTTCAGACGAGGA |
| Btmyosin-R | TTATTCAGGAATAATTGATTTT |
| Csmyosin-F | ATGAGCGACCTCAGCAAGAACG |
| Csmyosin-R | CTACGCCATCACCTTCTTCA |
| Dmmyosin-F | ATGGCTGATGTTCCAAAGCGTGA |
| Dmmyosin-R | CTATTTTAGCTGATCTGGTCT |
| Hlmyosin-F | ATGGCGGACCTCAAGCCCAATGA |
| Hlmyosin-R | CTACTTGTCCTTCTGCTCCTC |
| Pxmyosin-F | ATGACTGACCTCAGCAAAATCG |
| Pxmyosin-R | TTATTCTGCGTCATCCGGTTT |
| Semyosin-F | ATGAGCGACCTCAGCAAGAA |
| Semyosin-R | CTACGCCATCACCTTCTTCAG |
| Sfmyosin-F | ATGAGCGACCTCAGCAAGAA |
| Sfmyosin-R | TTAGCCCTCCGCGGGAGCTTC |
| Ttrmyosin-F | ATGGCTGACCAATTAACTGAA |
| Ttrmyosin-R | TTACTTAGAGGTCATCATGGTC |
| Turmyosin-F | ATGGCCGACCTTAAGCCCGCAG |
| Turmyosin-R | TTATTGAGCAGCAGCTTCCTCA |
| Temyosin-F | ATGGCTGACCAGTTAACTGAAGA |
| Temyosin-R | TCATTTGGATGTCATCATCGTT |
| RT-oemyosin-F | GCGGTGTCTACGAGGACTTC |
| RT-oemyosin-R | ATCATCATCCTCAGGGTCCA |

**Table S3. Primers used for vector construction and dsRNA synthesis**

| **Primers** | **Sequences (5’-3’)** |
| --- | --- |
| pBINPLUS-myosin-F | acccccggggtcgacggatccATGGCAGATGTCAGCAGCG |
| pBINPLUS-myosin-R | tctagttcatctagaggatccTTACAATACTGGCTTGATGTCATCTG |
| pBINPLUS-Nlmyosin-F | acccccggggtcgacggatccATGGCTGATGTGAGCAGTGAG |
| pBINPLUS-Nlmyosin-R | tctagttcatctagaggatccTTACAATACTGGCTTGATGTC |
| pBINPLUS-Asmyosin-F | acccccggggtcgacggatccATGGCCCACGATCTGAAGGAG |
| pBINPLUS-Asmyosin-R | tctagttcatctagaggatccTTACGGTGCGTGGTCACACA |
| pBINPLUS-Almyosin-F | acccccggggtcgacggatccATGAGCCAGGTCCGTAGGCCG |
| pBINPLUS-Almyosin-R | tctagttcatctagaggatccTCAAGGTATGATGGAGTCAG |
| pBINPLUS-Btmyosin-F | AacccccggggtcgacggatccTGGCCGATCTTTCAGACGAGGA |
| pBINPLUS-Btmyosin-R | tctagttcatctagaggatccTTATTCAGGAATAATTGATTTT |
| pBINPLUS-Csmyosin-F | acccccggggtcgacggatccATGAGCGACCTCAGCAAGAACG |
| pBINPLUS-Csmyosin-R | tctagttcatctagaggatccCTACGCCATCACCTTCTTCA |
| pBINPLUS-Dmmyosin-F | acccccggggtcgacggatccATGGCTGATGTTCCAAAGCGTGA |
| pBINPLUS-Dmmyosin-R | tctagttcatctagaggatccCTATTTTAGCTGATCTGGTCT |
| pBINPLUS-Hlmyosin-F | acccccggggtcgacggatccATGGCGGACCTCAAGCCCAATGA |
| pBINPLUS-Hlmyosin-R | tctagttcatctagaggatccCTACTTGTCCTTCTGCTCCTC |
| pBINPLUS-pxmyosin-F | acccccggggtcgacggatccATGACTGACCTCAGCAAAATCG |
| pBINPLUS-pxmyosin-R | tctagttcatctagaggatccTTATTCTGCGTCATCCGGTTT |
| pBINPLUS-Semyosin-F | acccccggggtcgacggatccATGAGCGACCTCAGCAAGAA |
| pBINPLUS-Semyosin-R | tctagttcatctagaggatccCTACGCCATCACCTTCTTCAG |
| pBINPLUS-Sfmyosin-F | acccccggggtcgacggatccATGAGCGACCTCAGCAAGAA |
| pBINPLUS-Sfmyosin-R | tctagttcatctagaggatccTTAGCCCTCCGCGGGAGCTTC |
| pBINPLUS-Ttrmyosin-F | acccccggggtcgacggatccATGGCTGACCAATTAACTGAA |
| pBINPLUS-Ttrmyosin-R | tctagttcatctagaggatccTTACTTAGAGGTCATCATGGTC |
| pBINPLUS-Turmyosin-F | acccccggggtcgacggatccATGGCCGACCTTAAGCCCGCAG |
| pBINPLUS-Turmyosin-R | tctagttcatctagaggatccTTATTGAGCAGCAGCTTCCTCA |
| pBINPLUS-Temyosin-F | acccccggggtcgacggatccATGGCTGACCAGTTAACTGAAGA |
| pBINPLUS-Temyosin-R | tctagttcatctagaggatccTCATTTGGATGTCATCATCGTT |
| pBINPLUS-myosin-1-81-F | acccccggggtcgacggatccATGGCAGATGTCAGCAGCG |
| pBINPLUS-myosin-1-81-R | tctagttcatctagaggatccACCGCAGTCCTTGTCCTTCC |
| pBINPLUS-myosin-82-161-F | acccccggggtcgacggatccGTCTACGAGGACTTCATTGA |
| pBINPLUS-myosin-82-161-R | tctagttcatctagaggatccTTACAATACTGGCTTGATGT |
| pBINPLUS-myosin-61-112-F | acccccggggtcgacggatccACAATCTCATTCGAAGAGTT |
| pBINPLUS-myosin-61-112-R | tctagttcatctagaggatccAGACAAGAGAATGTGTGAGA |
| pBINPLUS-myosin-45-93-F | acccccggggtcgacggatccCAGGGCCTCATTGAGAAACT |
| pBINPLUS-myosin-45-93-R | tctagttcatctagaggatccGTAGAGCTTCAAGCACTCAA |
| pBINPLUS-myosin-45-84-F | acccccggggtcgacggatccCAGGGCCTCATTGAGAAACT |
| pBINPLUS-myosin-45-84-R | tctagttcatctagaggatccCTCGTAGACACCGCAGTCCT |
| pBINPLUS-myosin-54-93-F | acccccggggtcgacggatccACCAAGAAGAAGGGTGAGAA |
| pBINPLUS-myosin-54-93-R | tctagttcatctagaggatccGTAGAGCTTCAAGCACTCAA |
| pBINPLUS-myosin-53-93-F | acccccggggtcgacggatccGGCACCAAGAAGAAGGGTGA |
| pBINPLUS-myosin-53-93-R | tctagttcatctagaggatccGTAGAGCTTCAAGCACTCAA |
| pBINPLUS-myosin-53-92-F | acccccggggtcgacggatccGGCACCAAGAAGAAGGGTGA |
| pBINPLUS-myosin-53-92-R | tctagttcatctagaggatccGAGCTTCAAGCACTCAATGA |
| dsRNA-myosin-F | GGATCCTAATACGACTCACTATAGGGACGTTGAAAAGTGCACCTT |
| dsRNA-myosin-R | GGATCCTAATACGACTCACTATAGGGCCAGGAATGGGATGTAGGG |
| dsRNA-GFP-F | TAATACGACTCACTATAGGGAGAATGAGTAAAGGAGAAGAACTTTTC |
| dsRNA-GFP-R | TAATACGACTCACTATAGGGAGATTTGTATAGTTCATCCATGCCATGT |

**Table S4. Primers used in qRT-PCR**

| **Primers** | **Sequences (5’-3’)** |
| --- | --- |
| qRT-myosin-F | GCGGTGTCTACGAGGACTTC |
| qRT-myosin-R | ATCATCATCCTCAGGGTCCA |
| qRT-Lsactin-F | TGGATTTGGCTGGACGAGAT |
| qRT-Lsactin-R | TCGGGCAATTCGTAGGACTT |
| qRTNbPR1-F | TGAGATGTGGGTCGATGAGA |
| qRTNbPR1-R | CGAGTTACGCCAAACCACTT |
| qRTNbPR2-F | AGGTGTTTGCTATGGAATGC |
| qRTNbPR2-R | TCTGTACCCACCATCTTGC |
| qRTNbPR3-F | TGGGGTTATTGCTGGCTTAG |
| qRTNbPR3-R | GGGTCATCCAAAACCAGAGA |
| qRTNbPR4-F | GGCCAAGATTCCTGTGGTAGAT |
| qRTNbPR4-R | CACTGTTGTTTGAGTTCCTGTTCCT |
| qRTNbCYP71D20-F | CCGCACCATGTCCTTAGAG |
| qRTNbCYP71D20-R | CTTGCCCCTTGAGTACTTGC |
| qRTNbWRKY7-F | CACAAGGGTACAAACAACACAG |
| qRTNbWRKY7-R | GGTTGCATTTGGTTCATGTAAG |
| qRTNbWRKY8-F | AACAATGGTGCCAATAATGC |
| qRTNbWRKY8-R | TGCATATCCTGAGAAACCATT |
| qRTNbAcre31-F | AATTCGGCCATCGTGATCTTGGTC |
| qRTNbAcre31-R | GAGAAACTGGGATTGCCTGAAGGA |
| qRT-Nbactin-F | CTGGGTTTGCTGGAGATGAT |
| qRT-Nbactin-R | CATCTCCCACGTAGGCATCT |
| qRT-OsMYC2-F | ACCAGATCCAGCACTTCGAG |
| qRT-OsMYC2-R | GCGAAGTCGGAGAAGTTGAG |
| qRT-GhMYC2-F | CCCAGCTTCCGTGCTTTATTTT |
| qRT-GhMYC2-R | GGAGGAAGGACTTTGATGGGT |
| qRT-GhJAZ3-F | TTCGCTTTGCCTTCGGTTATT |
| qRT-GhJAZ3-R | TGCCTACTCGTTGCCTGTTGT |
| qRT-OsPR1b-F | GATTAACTATGGAGGTATCCAAGC |
| qRT-OsPR1b-R | ACGTACGCCCGTGTGTATAAATAA |
| qRT-OsRBOHA-F | GCAGCAGATCACCGATAACA |
| qRT-OsRBOHA-R | GGCGTACTCTTCTGCTTGCT |
| qRT-OsBAK1-F | TTTCCTTGTGCATGCTAG |
| qRT-OsBAK1-R | GCATCTCCATAATTGATG |
| qRT-Osactin-F | CTGCGGGTATCCATGAGACT |
| qRT-Osactin-R | GCGACCACCTTGATCTTCAT |
| qRT-NbEDS1-F | GGTGGTCGTTGAGAATCCAG |
| qRT-NbEDS1-R | TCATCGTTACCACTTCATCA |
| qRT-NbBAK1-F | GGCTTTTGATCTTGCTCGAC |
| qRT-NbBAK1-R | CACCTCTCAGCTAGGCCATC |
| qRT-NbSOBIR1-F | CCGGGAAGTAATGGGAAGAT |
| qRT-NbSOBIR1-R | TATGTGCCAGTAGGGGAAGC |
| qRT-NbHSP90-F | GACTGCTCTTCTCACCTC |
| qRT-NbHSP90-R | TTCCATCTTGCTGCCTTC |
| qRT-NbNDR1-F | AGTGGTTTTCAGGGTGGATG |
| qRT-NbNDR1-R | ATTCGGGGAAGTCCAAAAGT |
| qRT-RSV-CP-F | TGCAGAAGGCAATCAATGACAT |
| qRT-RSV-CP-R | TGTCACCACCTTTGTCCTCAA |
